# Nanocellulose–MOF‐Derived Carbon Hybrid Aerogels with Hierarchical Micro/Nanostructures for Solar‐Driven Water Evaporation

**DOI:** 10.1002/advs.202516158

**Published:** 2025-10-24

**Authors:** Suji Lee, Kangyun Lee, Youngho Jeon, Yuri Seo, Seohyun Park, Youngsang Ko, Jungmok You

**Affiliations:** ^1^ Department of Convergent Biotechnology & Advanced Materials Science BK21 Interdisciplinary Program in IT‐Bio Convergence System and Graduate School of Green‐Bio Science Kyung Hee University 1732 Deogyeong‐daero, Giheung‐gu Yongin‐si Gyeonggi‐do 17104 South Korea; ^2^ NanoScience Technology Center University of Central Florida Orlando FL 32826 USA

**Keywords:** cellulose nanofiber, desalination, metal–organic framework (MOF)‐derived carbon, plasmonic nanoparticles, solar interfacial evaporation

## Abstract

Solar‐driven interfacial evaporation is a highly promising method for sustainable water purification. However, simultaneously achieving high photothermal efficiency and strong structural durability remains a considerable challenge. Here, a hierarchically engineered bilayer evaporator is introduced, fabricated entirely through aqueous processing. This device combines a vertically aligned nanocellulose layer for water transport with a plasmonically enhanced, metal–organic framework (MOF)‐derived carbon layer for photothermal conversion. Using a sequential ice‐templating technique, both layers feature anisotropic microchannels that facilitate rapid, capillary‐driven water transport and efficient vapor release, while providing robust interfacial adhesion without requiring additional binders. The photothermal layer, made of ZIF‐8‐derived porous carbon uniformly decorated with gold nanoparticles (AuNPs), achieves broadband solar absorption of 95.9% and efficient localized heating through plasmonic effects. At an optimal AuNP loading of 40 wt%, the evaporator reaches a peak water evaporation rate of 2.36 kg m^−2^ h^−1^ and an apparent solar‐to‐vapor efficiency of 119% under 1 sun illumination. The system performs well in highly saline water, offers excellent self‐cleaning, and is both fully biodegradable and scalable. This work introduces an eco‐friendly and scalable platform for efficient solar vapor generation with potential in seawater desalination, off‐grid freshwater supply for remote and disaster‐affected regions, and sustainable wastewater treatment and reuse.

## Graphical abstract

A biodegradable, cellulose‐based bilayer solar evaporator is fabricated entirely via aqueous, sequential ice‐templating. The hybrid structure combines vertically aligned cellulose nanofibers for rapid water transport with a metal‐orgnic framework (MOF)‐derived porous carbon layer decorated with plasmonic nanoparticles for enhanced photothermal conversion. This eco‐friendly platform enables scalable fabrication and efficient clean‐water production from saline sources.

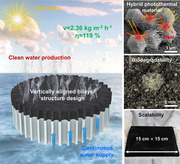

## 1 Introduction

The rapid increase in the global population, coupled with climate change and environmental pollution, has caused a critical shortage of freshwater resources worldwide.^[^ ^1^ ^]^ By 2050, nearly 30% of the population is projected to face severe drinking water scarcity.^[^ ^2^, ^3^ ^]^ Among the available strategies, solar‐driven desalination has emerged as a promising approach, as it harnesses abundant solar energy and the vast seawater resources covering more than 70% of Earth's surface.^[^ ^4^, ^5^, ^6^ ^]^ Unlike conventional bulk heating, solar interfacial evaporation concentrates solar energy at the air–water interface, thereby minimizing heat loss and enabling superior photothermal conversion efficiency.^[^ ^5^, ^7^, ^8^ ^]^ These systems typically include two key components: a support structure that supplies water to the evaporation surface, and a photothermal layer that absorbs solar energy and converts it into heat, driving water evaporation.^[^ ^4^, ^5^, ^8^, ^9^, ^10^ ^]^ The support structure must provide a continuous water flow to the evaporation interface through a porous, hydrophilic design while preventing heat loss from the interface to the bulk water below. Additionally, the photothermal layer plays a critical role in determining the overall evaporation efficiency. To efficiently convert absorbed light into heat, the material must have a high absorption rate over a broad wavelength range, spanning from ultraviolet (UV) to near‐infrared (NIR). However, many reported solar interfacial evaporators still face critical bottlenecks that hinder their practical deployment. For example, synthetic polymer‐based supports, while capable of facilitating water transport, lack biodegradability and pose long‐term environmental hazards through micro‐ and nano‐plastic pollution.^[^ ^11^, ^12^ ^]^ In addition, photothermal nanomaterials are often prone to aggregation, uneven distribution, and weak interfacial adhesion, leading to unstable evaporation performance and structural degradation over time.^[^ ^13^ ^]^ Importantly, stable interfacial bonding, efficient light absorption, thermal confinement, rapid water transport, and robust photothermal–substrate integration are all essential to sustain high efficiency and long‐term durability in solar interfacial evaporators.^[^ ^4^, ^5^, ^8^, ^9^, ^10^ ^]^

Various photothermal materials have been explored for solar interfacial evaporation, including plasmonic metals (e.g., Au, Ag), semiconductors (e.g., TiO_2_, Cu_2_O), conductive polymers (e.g., polyaniline, polythiophene), and carbon‐based materials (e.g., graphene, carbon nanotubes, carbon black).^[^ ^14^, ^15^, ^16^, ^17^, ^18^, ^19^, ^20^, ^21^ ^]^ Among these, carbon‐based materials stand out for their broadband light absorption, chemical and thermal stability, and scalability.^[^ ^22^, ^23^ ^]^ In particular, metal‐organic framework (MOF)‐derived porous carbons offer uniform structures, high surface area, and tunable hierarchical porosity, which enhances light scattering, photon trapping, and water transport, while also providing abundant sites for functional nanomaterial incorporation to further boost photothermal efficiency.^[^ ^24^, ^25^, ^26^, ^27^, ^28^, ^29^ ^]^

Cellulose, a naturally derived biopolymer recognized for its biodegradability and sustainability, is an excellent candidate for water transport structures. Its abundant hydroxyl groups (–OH) provide strong hydrophilicity, enabling rapid and continuous water absorption.^[^ ^30^, ^31^, ^32^ ^]^ Specifically, cellulose nanofibers (CNFs) can be formed into a porous matrix that combines high mechanical strength and flexibility. Additionally, their naturally low thermal conductivity helps retain heat near the evaporation interface, enhancing efficiency.^[^ ^32^ ^]^ In contrast, cellulose‐based supports are environmentally friendly and harmless to ecosystems, making them a sustainable alternative for future solar evaporators.^[^ ^33^ ^]^

Our group previously developed a highly efficient solar interfacial evaporator by directly forming a carbon layer on cellulose aerogel through a simple and rapid CO_2_ laser carbonization process.^[^ ^32^ ^]^ Although this system showed excellent photothermal performance, its reliance on a polydimethylsiloxane coating to ensure mechanical stability and buoyancy compromised its biodegradability, preventing it from being fully bio‐based. Additionally, the laser‐induced carbonization often results in nonuniform structures and limited thickness control, which can adversely affect light absorption and thermal management in the evaporator.

In this study, we fabricate a double‐layer solar evaporator using sequential ice templating to integrate a CNF aerogel (CA) support with a gold nanoparticle (AuNP)‐loaded porous ZIF‐8 carbon (ZC) photothermal layer. To address the aforementioned challenges, which include weak interfacial adhesion between layers, limited structural durability, the trade‐off between photothermal efficiency and biodegradability, and difficulties in achieving scalability in fabrication processes, we developed a hierarchically engineered, biomass cellulose‐based solar evaporator entirely fabricated through an aqueous, sequential ice‐templating process. This design incorporates a vertically aligned nanocellulose support layer with a uniformly dispersed photothermal carbon layer, ensuring strong interfacial adhesion, high evaporation efficiency, and excellent environmental sustainability. The CA's CNF matrix is chemically cross‐linked with glutaraldehyde (GA) to improve mechanical stability. The ZIF‐8 precursor is synthesized in water, avoiding organic solvents, and thermally converted into a nitrogen‐doped, partially graphitized porous carbon framework. Importantly, a hybrid photothermal material, AuNP‐loaded ZC (AZC), is created via in situ growth of AuNPs in the porous carbon matrix. Importantly, the aqueous AZC dispersion exhibited excellent compatibility with the hydrophilic CNFs, enabling the easy preparation of AZC–CNF composite dispersions through simple mixing. This strong compatibility facilitated the fabrication of the evaporator by sequential ice templating of the cross‐linked CNF dispersion followed by the AZC–CNF composite dispersion, ensuring integrated structural coherence and functional synergy. The process produced vertically aligned microchannels that promote directional water transport and speed up moisture removal. Additionally, separating the water transport support from the photothermal layer reduced heat loss to the bulk water. The resulting AZC–CA/CA evaporator achieved high evaporation rates, superior desalination performance, and long‐term operational stability. Furthermore, outdoor field tests under natural sunlight confirmed its stable and durable performance, demonstrating the practical applicability of this eco‐friendly and scalable approach. This study enhances the understanding of nanofibrous biopolymers as fundamental building blocks and their assembled macro composites, paving the way for the development of high‐performance, sustainable solar evaporators.

## 2 Results and Discussion

### 2.1 Fabrication and Hierarchical Design of AZC‐CA/CA Evaporator

**Figure 1:**
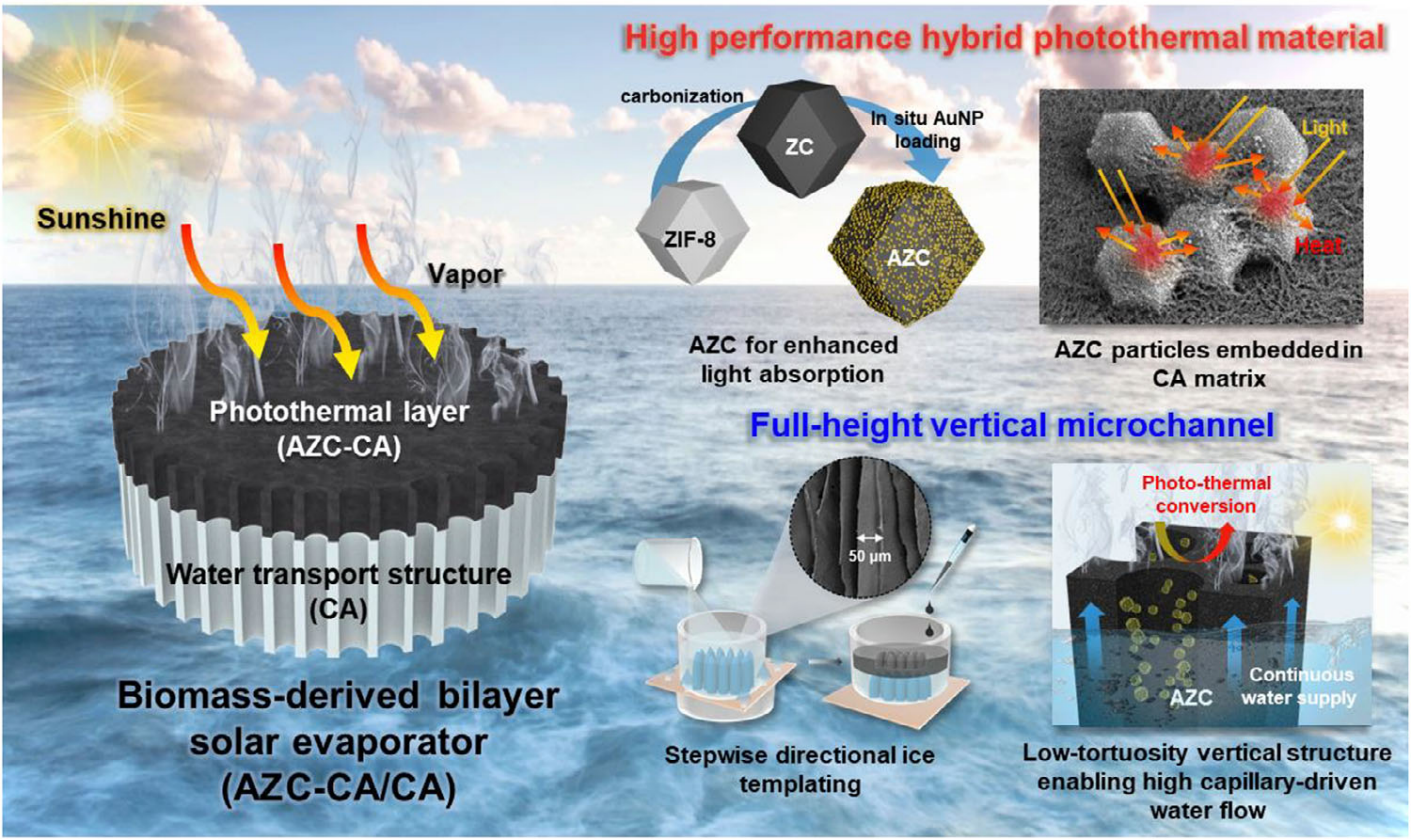
Schematic illustration of the bilayer AZC–CA/CA solar evaporator fabricated through sequential ice templating. The bottom layer, made of pure CNF‐based CA, contains vertically aligned water channels that facilitate efficient water transport. The top layer, a hybrid AZC‐embedded CNF photothermal layer (AZC–CA), provides effective solar‐to‐heat conversion.

**Figure** **1** shows the bilayer solar evaporator, consisting of a vertically aligned CA water transport layer and an upper hybrid AZC–CA photothermal layer. This sustainable evaporator is fabricated via a scalable, sequential ice‐templating process using CNF and AZC/CNF dispersions, which incorporate hybrid plasmonic carbon particles into a biomass‐derived framework. Both layers feature a vertically aligned, porous architecture created through directional freezing, enabling rapid upward water transport and low‐resistance vapor escape. Unlike traditional photothermal materials, the hybrid AZC particles provide broadband solar absorption and localized plasmonic heating while remaining stably immobilized in the CNF matrix. The highly porous AZC particles enhance solar absorption through multiple scattering and localized plasmonic effects, resulting in efficient and stable interfacial evaporation. This integrated structural–compositional design combines effective photothermal conversion with sustainable material processing, thereby delivering superior evaporation performance over conventional approaches.

**Figure 2:**
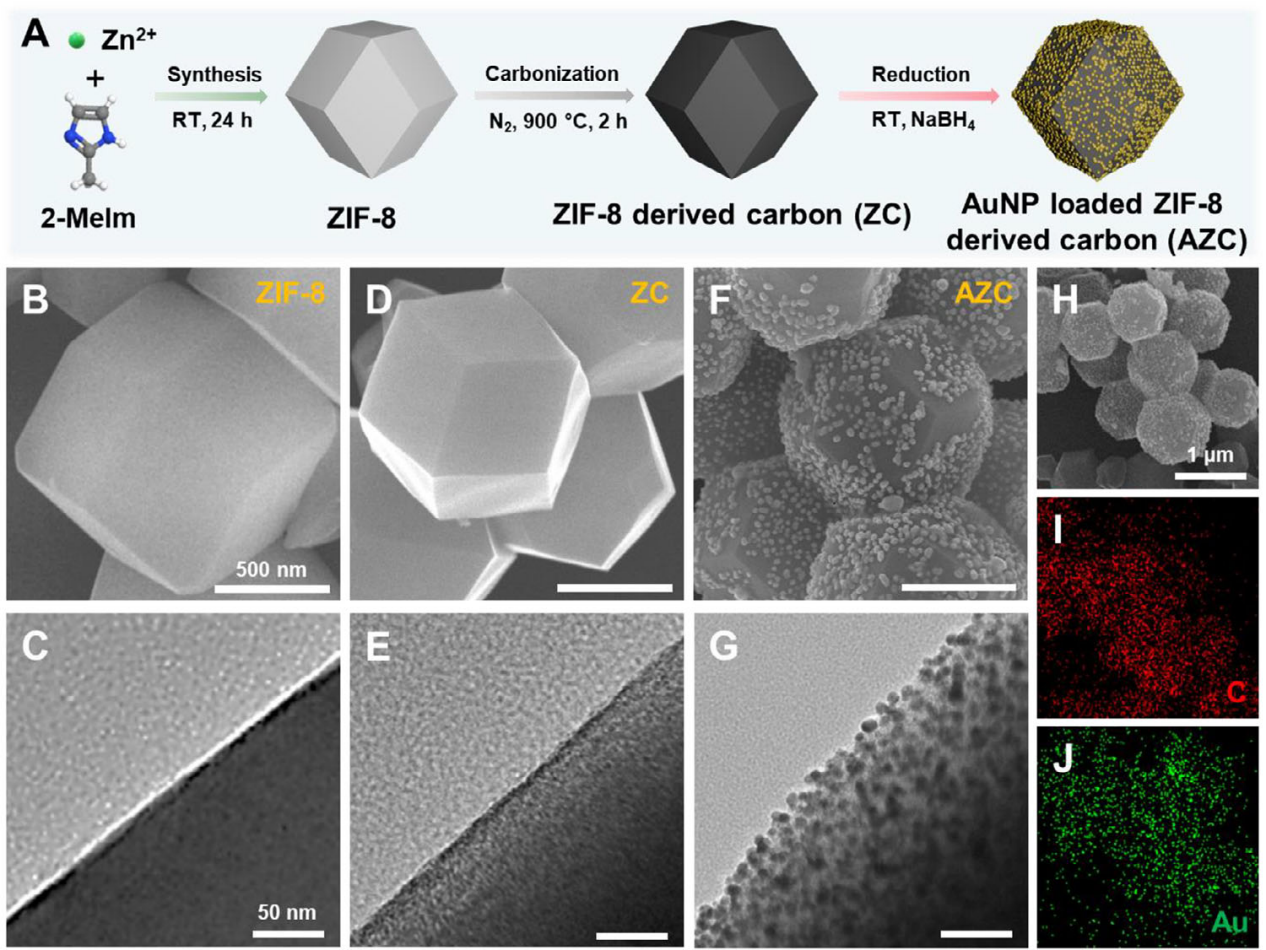
A) Schematic illustration of the preparation of AZC. Field emission scanning electron microscopy (FE‐SEM) and field emission transmission electron microscopy (FE‐TEM) images of B,C) ZIF‐8, D,E) ZC, and F,G) AZC powders. H–J) Energy‐dispersive spectroscopy (EDS) elemental mapping images of AZC.

**Figure** **2**A shows the synthesis process of AZC. First, ZIF‐8 particles were formed by coordinating zinc ions (Zn^2+^) with 2‐methylimidazole (2‐MeIm) at room temperature. These particles were then carbonized under nitrogen at 900 °C to produce porous ZC. Subsequently, AuNPs were uniformly deposited on the ZC surface through in situ reduction using sodium borohydride (NaBH_4_), creating AZC with enhanced light absorption and plasmonic features. Electron microscopy characterized the morphological evolution, showing ZIF‐8 nanoparticles with a consistent rhombic dodecahedral shape and smooth surfaces (Figure 2B,C). This morphology was consistent with dynamic light scattering (DLS) results, which indicated a narrow size distribution centered ≈1.2 µm (Figure S1A, Supporting Information). After carbonization, the ZC particles slightly shrank to ≈800–900 nm, as confirmed by a DLS shift toward smaller diameters (Figure S1B, Supporting Information), while maintaining their polyhedral shape (Figure 2D,E). This size reduction was caused by structural shrinkage owing to the decomposition of organic linkers and partial evaporation of zinc at temperatures above 550 °C. These changes led to a roughened surface and a carbon scaffold exhibiting a high specific surface area of 793 m^2^ g^−1^, as measured by Brunauer–Emmett–Teller (BET) analysis (Figure S1D, Supporting Information). Then, AuNPs sized between 2 and 8 nm were uniformly anchored onto the ZC surface (Figure 2F,G). The DLS profile of the AZC particles showed a slight increase in hydrodynamic diameter to ≈950 nm (Figure S1C, Supporting Information), likely caused by surface roughening and AuNP decoration, while preserving a unimodal distribution with no signs of aggregation. BET analysis indicated a surface area of ≈371 m^2^ g^−1^ for AZC, which is lower than that of ZC (≈793 m^2^ g). These findings demonstrate that AuNPs were successfully deposited on the surface and partially embedded in the pores of ZC, confirming their uniform integration into the porous carbon structure. The uniform nucleation and growth of AuNPs were attributed to the high porosity, surface defects, and nitrogen functionalities of ZC, which offered abundant coordination sites for Au precursor adsorption and localized reduction.^[^ ^34^, ^35^, ^36^ ^]^ These structural characteristics promote a homogeneous AuNP distribution and prevent aggregation by spatial confinement and strong interfacial interactions with the carbon matrix. Adjusting the Au precursor concentration allows effective control over AuNP loading on the ZC surface. In the A_0.5_ZC sample, AuNPs showed a low loading density and were sparsely, individually distributed on the carbon matrix (Figure S2A,B, Supporting Information). Increasing the Au precursor concentration to form A_1_ZC led to a higher amount of AuNPs, which remained uniformly distributed with minimal aggregation (Figure S2C,D, Supporting Information). In contrast, the AZC sample exhibited a considerably greater AuNP density, with nanoparticles densely packed and forming a continuous coating on the carbon surface (Figure S2E,F, Supporting Information). This uniform distribution in AZC was further validated by EDS elemental mapping analysis (Figure 2H–J). Such a consistent and continuous AuNP coating is expected to facilitate stable and efficient light‐to‐heat conversion, a crucial factor for ensuring reliable performance in solar‐driven evaporation. Accordingly, the controlled deposition of AuNPs onto porous ZC not only maximizes broadband light absorption but also ensures stable plasmonic heating, thereby directly linking nanoscale structural design to enhanced macroscopic evaporation efficiency.

**Figure 3:**
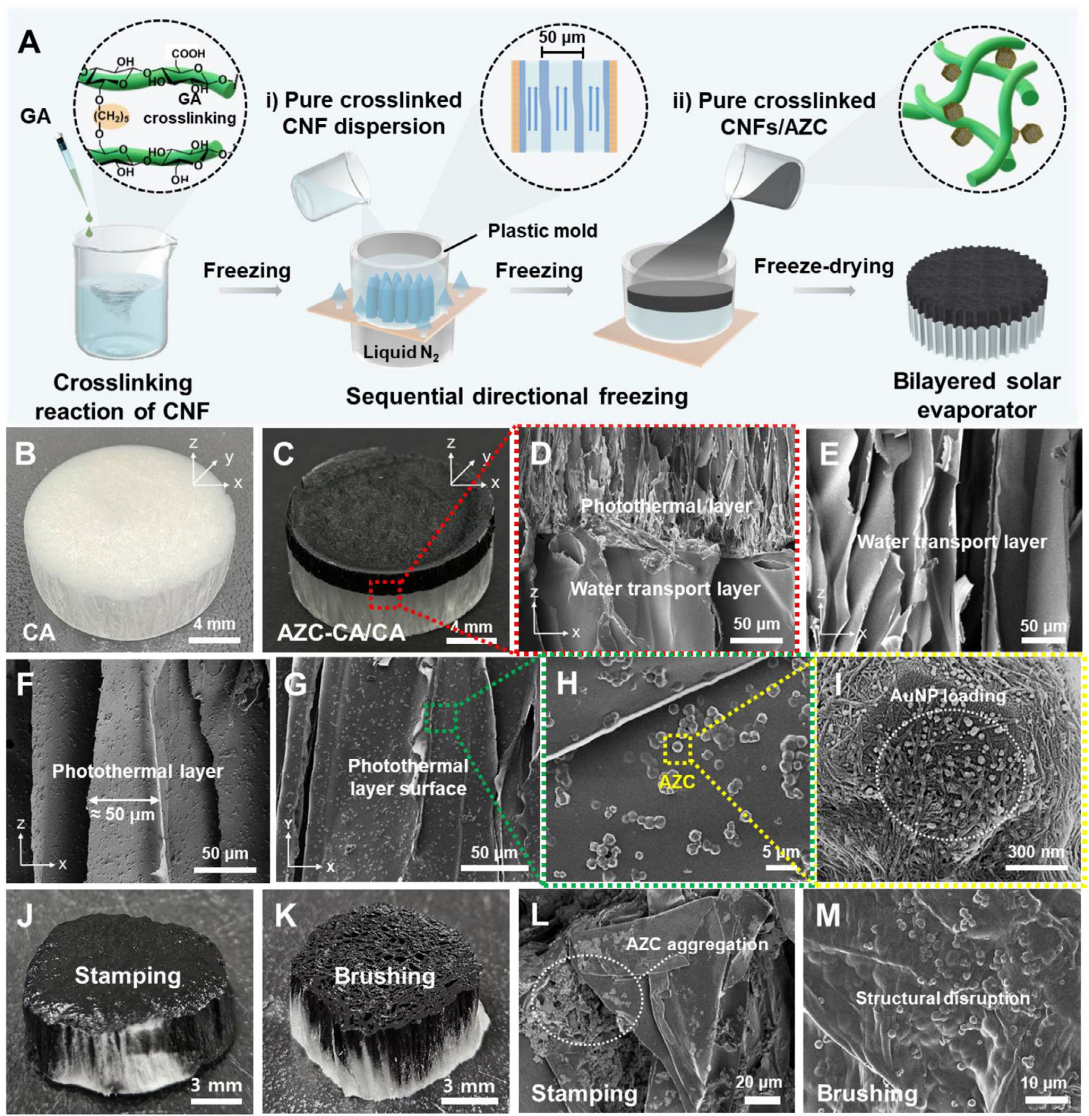
A) Schematic illustration of AZC–CA/CA preparation: 2,2,6,6‐tetramethylpiperidin‐1‐oxyl (TEMPO)‐CNFs were chemically cross‐linked with GA, followed by the addition of AZC particles to the cross‐linked CNF dispersion. i) The cross‐linked CNF dispersion was poured into a copper plate using a plastic mold, then subjected to ice templating under liquid nitrogen. ii) The AZC/cross‐linked CA dispersion was applied to the prefrozen surface of the water transport layer, forming a bilayer structure. Photographic images show B) CA and C) AZC–CA/CA evaporators. D) Cross‐sectional SEM image highlights the interface between the photothermal AZC layer and the water transport CA layer. E) FE‐SEM image shows the structure of the water transport CA layer. F–I) Magnified SEM images of the photothermal AZC–CA layer reveal vertically aligned CA channels and uniform AZC particle distribution. Photographs and cross‐sectional FE‐SEM images of AZC–CA/CA evaporators fabricated by J,L) stamping and K,M) brush coating methods.

**Figure** **3**A shows the fabrication process and structural design of a bilayer solar evaporator. Cross‐linking between CNFs and GA occurs through the formation of acetal or hemiacetal linkages between the hydroxyl groups (–OH) of CNFs and the aldehyde groups (–CHO) of GA.^[^ ^37^ ^]^ This reaction creates a stable 3D network in the cellulose matrix, enhancing the mechanical strength and structural integrity of the evaporator. Subsequently, photothermal AZC particles are incorporated into the cross‐linked CNF dispersion. Because both cross‐linked CNFs and AZC are easily dispersible in water, the mixture exhibits excellent colloidal stability, which reduces particle aggregation and ensures a uniform dispersion of AZC throughout the CNF matrix. To construct the bilayer structure, the pure cross‐linked CNF dispersion was first directionally frozen using the ice‐templating method to create a vertically aligned water transport layer (Figure 3A‐i). Next, the AZC/cross‐linked CNF dispersion was poured onto the prefrozen CNF surface. The subsequent freezing and drying steps formed the photothermal top layer over the water‐transport layer (Figure 3A‐ii). This controlled ice‐templating strategy not only ensured strong interfacial adhesion between layers without needing extra adhesives but also enabled the creation of continuous, vertically aligned microchannels throughout the evaporator. These anisotropic channels were engineered to facilitate directional water transport in the structure and allow efficient water vapor escape from the photothermal layer, considerably improving the efficiency of solar‐driven interfacial evaporation. As evidenced by the photographs of the pure CA and AZC–CA/CA evaporators, the AZC–CA/CA clearly exhibits a bilayer structure, featuring a uniform photothermal top layer and a water‐transporting bottom layer (Figure 3B,C). As shown in Figure 3D, cross‐sectional SEM images at the interface demonstrated stable adhesion between the photothermal AZC–CA layer and the water‐transport CA layer, with no delamination observed. This robust interfacial adhesion was further validated by mechanical adhesion tests (3M tape and compression tester) and immersion–sonication stability tests, all of which showed no separation between the two layers (Figure S3, Supporting Information). The water‐transport CA layer featured vertically aligned microchannels ≈50 µm in diameter, enabling efficient, directional water transport (Figure 3E). At the interface, the pore size appeared slightly different between the water‐transport and photothermal layers (Figure 3D). This variation originates from the sequential ice‐templating process, where the lower layer is frozen first and the upper dispersion subsequently undergoes freezing.^[^ ^38^, ^39^ ^]^ Cross‐sectional SEM images further confirmed that both layers contained vertically aligned channels with only minor pore size differences across their thickness (Figure S4, Supporting Information). Specifically, smaller pores were observed near the down regions, where ice crystals nucleated and grew rapidly, whereas slightly larger channels appeared toward the upper regions as ice growth stabilized. These pore size variations can be attributed to differences in ice nucleation and growth dynamics during directional freezing. Figure 3F,G shows these vertically aligned microchannels in CA, along with a uniform AZC distribution in the photothermal AZC–CA layer. Figure 3H confirms that AZC particles were stably and uniformly embedded in the CA matrix. Importantly, the magnified FE‐SEM images (Figure 3I) clearly show the in situ‐synthesized AuNPs in ZC inside the AZC–CA layer. Unlike the AZC–CA/CA evaporator, the ZC–CA/CA evaporator showed no presence of AuNPs, although its overall morphological structure was similar (Figure S5, Supporting Information). Compared to conventional stamping or brush coating methods typically used to apply photothermal nanomaterials onto water transport structures, the sequential freezing process used here provided superior interfacial adhesion and structural continuity. To highlight the advantages of the sequential freezing method, photothermal evaporators were also prepared by applying AZC materials onto the CA surface using traditional stamping and brush coating techniques for comparison. As shown in Figure 3J,K, the AZC–CA/CA evaporators produced by conventional methods exhibited structural collapse and uneven AZC distribution on the CA matrix, which may impair evaporation performance. Magnified SEM images (Figure 3L,M) show substantial channel misalignment owing to structural collapse, as well as uneven AZC loading and aggregation. These results demonstrate that traditional coating techniques lack the precision needed for uniformly distributing photothermal materials in water transport microstructures. These structural deficiencies resulted in inferior evaporation performance. IR thermal imaging revealed that the surface temperature of the conventional evaporators was ≈5.4 °C lower than that of the sequentially frozen AZC–CA/CA evaporator, indicating weaker solar‐to‐thermal conversion efficiency (**Figure** **5**B; Figure S6A, Supporting Information, discussed later). In addition, solar evaporation tests under 1 sun illumination confirmed that the conventionally fabricated evaporators exhibited lower and less reproducible evaporation rates, with average values of 1.25 ± 0.22 kg m^−2^ h^−1^ for the stamping method and 1.05 ± 0.15 kg m^−2^ h^−1^ for the brush‐coating method. Both values are significantly lower than that of our sequentially frozen AZC–CA/CA evaporator (2.36 ± 0.25 kg m^−2^ h^−1^, see Figure 5F; Figure S6B, Supporting Information, discussed later). In contrast, our sequential freezing process improves photothermal conversion efficiency at the evaporation interface while preserving structural stability and durability during extended use. This distinctive methodology integrates robustness with superior evaporation performance, clearly surpassing conventional coating‐based fabrication approaches.

### 2.2 Chemical Composition, Mechanical Stability, and Water Transport Properties

**Figure 4:**
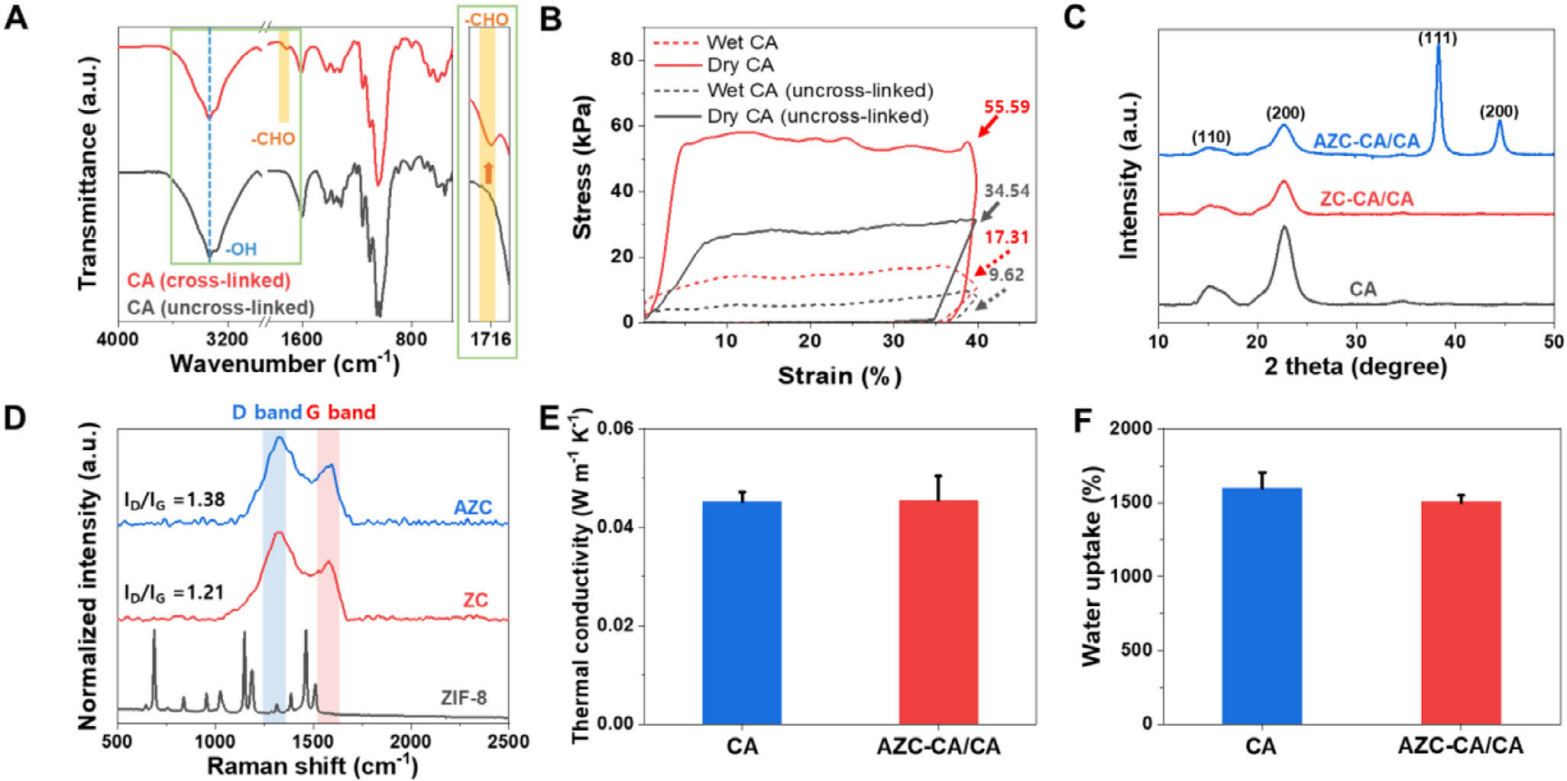
A) FT–IR spectra and B) Compression test data of cross‐linked CA and uncross‐linked CA. C) XRD patterns of the CA, ZC‐CA/CA, and AZC‐CA/CA evaporator. D) Raman spectra of the ZIF‐8, ZC, and AZC powder. E) Thermal conductivities of CA and AZC‐CA/CA evaporator. F) Weight increment of CA and AZC‐CA/CA evaporator after water immersion for 3 h.

To investigate the effect of GA cross‐linking, Fourier‐transform infrared spectroscopy (FT–IR) analysis and compression testing were performed on both uncross‐linked CA and cross‐linked CA. In the FT–IR spectra (**Figure** **4**A), uncross‐linked CA exhibited characteristic peaks for –OH stretching (≈3340 cm^−1^), C–H stretching (≈2900 cm^−1^), and C–O–C/C–OH bending (≈1050–1150 cm^−1^). After cross‐linking, a new –CHO peak appeared at 1716 cm^−1^, and the intensity of the –OH peak decreased, indicating the consumption of hydroxyl groups and the formation of covalent bonds between GA and CNF. To evaluate how GA cross‐linking affects mechanical stability, compression tests were performed on both uncross‐linked and cross‐linked CA samples in dry and water‐saturated conditions (Figure 4B). The cross‐linked CA exhibited enhanced structural integrity at 40% strain, registering a compressive strength of 55.59 kPa in the dry state and 17.31 kPa in the wet state. These values are consistent with cellulose‐based aerogel evaporators previously applied in solar desalination, indicating sufficient mechanical robustness for practical use.^[^ ^40^, ^41^ ^]^ This considerably outperformed the uncross‐linked CA, which showed strengths of 34.54 and 9.62 kPa, respectively. Such results clearly demonstrate that GA cross‐linking substantially improves mechanical stability in both dry and wet conditions. Moreover, it provides a controllable level of strength even when hydrated—a key advantage because cellulose aerogels are typically fragile in wet environments. This confirms that GA cross‐linking effectively mitigates this fragility, providing reliable structural support during continuous evaporation.

For further insights into the structural and compositional features of AZC incorporation, X‐ray diffraction (XRD) and Raman spectroscopy were performed. XRD analysis revealed that all samples consistently retained the crystalline structure of cellulose I, showing broad diffraction peaks at 2θ = 15.6° and 22.5° (Figure 4C). These correspond to the (110) and (200) planes of CNF, indicating that the incorporation of AZC into the CA matrix did not disrupt the cellulose's overall crystallinity. Complementing this, new diffraction peaks at 2θ = 38.2° and 44.4° were observed in the AZC–CA/CA samples, corresponding to the (111) and (200) planes of face‐centered cubic (fcc) AuNPs.^[^ ^42^, ^43^ ^]^ Figure 4D shows that ZIF‐8 exhibits sharp Raman peaks characteristic of imidazole rings, which disappear in ZC owing to carbonization. Raman analysis of both ZC and AZC showed distinct D (≈1350 cm^−1^, representing disordered carbon) and G (≈1580 cm^−1^, representing graphitic carbon) bands. A slightly higher I_D_/I_G_ ratio for AZC (1.38) compared to ZC (1.21) confirms greater defect incorporation resulting from AuNP presence. This defect‐rich structure is advantageous for photothermal performance because it broadens light absorption and enhances non‐radiative heat conversion.^[^ ^44^ ^]^ Thus, AZC incorporation preserves the intrinsic cellulose crystalline framework while simultaneously introducing plasmonic and defect‐rich carbon features, providing a synergistic enhancement in broadband light harvesting and heat generation.

In solar‐driven interfacial evaporators, thermal conductivity and water absorption properties are critical performance indicators. Figure 4E shows that both CA (0.045 W m^−1^ K^−1^) and AZC–CA/CA (0.053 W m^−1^ K^−1^) exhibited remarkably low thermal conductivities, attributable to the intrinsically low conductivity of cellulose and their hierarchically porous architectures, which hinder phonon transport through multiple scattering interfaces and air‐filled channels.^[^ ^45^ ^]^ Additional measurements of ZC–CA/CA evaporators with varying ZC loadings revealed thermal conductivities within a narrow range of 0.055–0.059 W m^−1^ K^−1^, indicating that heat transport remains governed by the cellulose aerogel framework and its porous structure rather than the ZC content (Figure S8B, Supporting Information). Although the AZC layer incorporates carbon, which is intrinsically more conductive, its contribution is negligible due to the small volume fraction relative to the CNF matrix. Consequently, the overall thermal conductivity is dominated by the porous cellulose framework, which minimizes heat dissipation into the bulk water and enables localized heat confinement at the evaporation interface. This regulation of interfacial heat transfer by the hierarchically porous, low‐conductivity cellulose framework provides the fundamental design principle linking structural architecture to enhanced photothermal efficiency.

This low thermal conductivity is advantageous for minimizing heat loss at the evaporation interface and facilitating localized heat confinement during solar‐driven water evaporation. Regarding water absorption, Figure 4F shows that both CA and AZC–CA/CA exhibit high capacities, reaching ≈1500%. This high absorption is attributed to the numerous nano‐ and microchannels in the aerogel, which facilitate rapid and efficient water transport. Moreover, CA demonstrated exceptional wettability, evidenced by complete water droplet absorption within 0.05 s (Figure S7, Supporting Information), stemming from its highly porous structure and inherent hydrophilic nature. Although the presence of photothermal particles in ZC–CA/CA and AZC–CA/CA resulted in a minor delay in absorption (≈0.14 and 0.15 s, respectively), rapid wettability was retained, ensuring continuous and directional water replenishment essential for sustained high evaporation rates. Overall, GA cross‐linking imparts mechanical robustness under wet conditions, the low thermal conductivity confines heat at the evaporation interface, and ultrafast water absorption guarantees continuous mass supply; together, these synergistic features ensure long‐term stability and efficient interfacial heat–mass transfer during solar‐driven evaporation.

### 2.3 Enhanced Photothermal Conversion and High‐Performance Desalination

**Figure 5:**
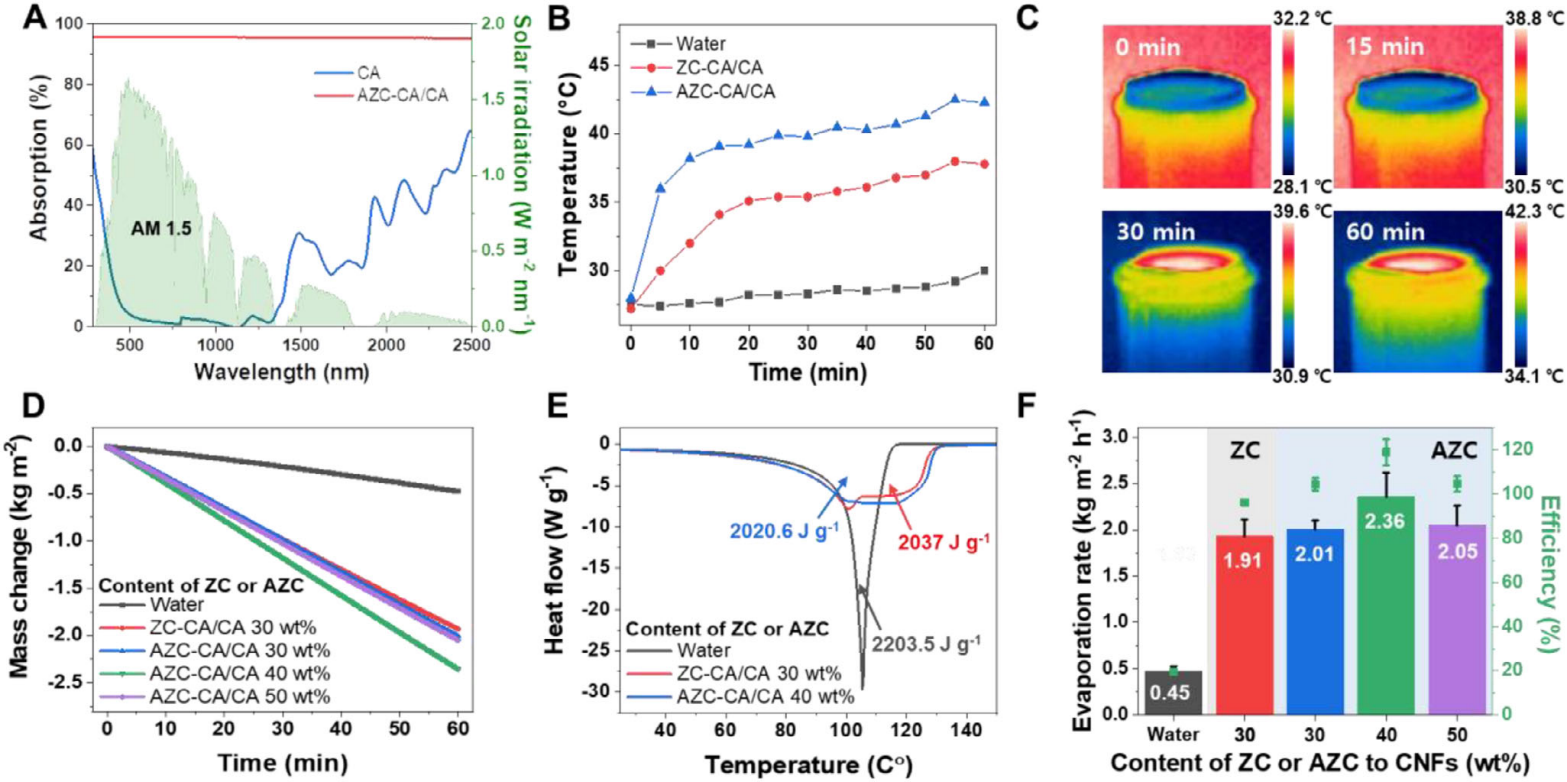
A) UV–vis–NIR absorption spectra of CA and AZC‐CA/CA evaporator. B) Photothermal heat generation of pure water, ZC‐CA/CA, and AZC‐CA/CA evaporator. C) IR thermal images of AZC‐CA/CA. D) Mass change and E) DSC analysis of bulk water, ZC‐CA/CA, and AZC‐CA/CA with different contents of ZC or AZC to CNFs. F) Evaporation rates and efficiencies from bulk water, ZC‐CA/CA, and corresponding AZC‐CA/CA samples. All solar vapor generation measurements were performed under 1 sun illumination.

Figure 5 shows the photothermal evaporation performance of the solar evaporators. To thoroughly investigate the contribution of AZC hybrid materials, a control evaporator was specifically fabricated using only the ZC photothermal component (without AuNPs). As shown in Figure 5A, the AZC–CA/CA evaporator exhibited high light absorption across the solar spectrum, reaching 95.9%, in contrast to only 7.5% for CA. For reference, ZC–CA/CA evaporators with 30, 40, and 50 wt% ZC relative to CNFs, as well as pure ZC powder, showed solar‐weighted absorption values of 94.5, 94.2, 93.7, and 91%, respectively (Figure S8A, Supporting Information). These results demonstrate that excessive ZC loading leads to increased optical attenuation and backscattering, which in turn limits light transmission and reduces evaporation performance. The addition of AuNPs further enhanced absorption to 95.9% by inducing multiple scattering and strong localized plasmonic heating on the porous ZC surface. In contrast, conventional carbon materials with relatively smooth surfaces lack such plasmonic effects, resulting in weaker light–matter interactions. Thus, the synergistic combination of broadband absorption from the carbon matrix and plasmonic resonance from AuNPs enables superior light harvesting. Consistently, photothermal heating profiles and IR thermal images (Figure 5B,C) clearly demonstrate that the AZC–CA/CA evaporator attains a much faster and higher increase in surface temperature under 1 sun illumination than both pure water and the ZC–CA/CA evaporator. Under 1 sun illumination, the pure water exhibited a modest temperature increase of only 6 °C over 1 h. The ZC–CA/CA evaporator showed a more pronounced thermal response, with its temperature increasing by 12 °C to reach 38.2 °C after the same duration. Critically, the surface temperature of the AZC–CA/CA evaporator rapidly increased by 15 °C in the initial 5 min, reaching 43.2 °C after 1 h. After these observations, we investigated the evaporation performance of both ZC–CA/CA and AZC–CA/CA evaporators at different ZC and AZC loading percentages (wt% relative to CNFs) (Figure 5D–F; Figure S8C,D, Supporting Information). Within the 10–40 wt% ZC loading range, the evaporation rate steadily improved from 1.49 ± 0.09 to 1.91 ± 0.05 kg m^−2^ h^−1^, confirming adequate photothermal activity. In contrast, ZC–CA/CA with 50 wt% ZC loading exhibited reduced performance, primarily due to impaired light penetration caused by excessive carbon accumulation. By integrating the evaporation rate data with the optical and thermal measurements, a clear quantitative correlation emerges: increasing ZC loading from 30 to 40 wt% enhances light absorption and evaporation efficiency, whereas excessive loading at 50 wt% lowers performance due to limited light transmission rather than changes in thermal insulation. In parallel, the evaporation performance of AZC–CA/CA was assessed across varying AZC contents, from 30 to 50 wt% (Figure 5D–F). Crucially, all AZC–CA/CA evaporators consistently exhibited superior evaporation performance when compared to the optimized ZC–CA/CA evaporators. Notably, the 40 wt% AZC–CA/CA sample achieved the highest water evaporation rate of 2.36 ± 0.25 kg m^−2^ h^−1^. Differential scanning calorimetry (DSC) analysis was further employed to quantify the intermediate water content in ZC–CA/CA and AZC–CA/CA samples (Figure S9, Supporting Information). The AZC–CA/CA samples contained a larger fraction of intermediate water, which reduces the effective enthalpy of evaporation and thus contributes to their enhanced evaporation performance. To further understand the energy conversion from photothermal heating into water vapor, the enthalpy of water evaporation was experimentally determined via DSC for pure water, ZC–CA/CA, and AZC–CA/CA evaporators (Figure 5E). The vaporization enthalpies determined for pure water, 30 wt% ZC–CA/CA, and 40 wt% AZC–CA/CA were 2203.5, 2037.0, and 2020.6 J g^−1^, respectively. Notably, the values obtained from the bilayer evaporators were consistently lower than those of pure water, even under identical experimental conditions. This observed reduction is attributed to the formation of intermediate and bound water states in the porous cellulose structure.^[^ ^46^, ^47^ ^]^ Specifically, the hydrophilic functional groups on the CNFs weaken the hydrogen bonds between water molecules. This, in turn, promotes the formation of intermediate water, a state where water molecules are loosely bound to the matrix, consequently exhibiting a reduced enthalpy of vaporization. Based on these experimentally determined enthalpy values and the net evaporation rates (calculated from the difference between illuminated and dark conditions, as detailed in Figure S10, Supporting Information), the apparent solar‐to‐vapor conversion efficiencies were subsequently calculated (Figure 5F). Table S1 (Supporting Information) summarizes all parameters used for these efficiency calculations, including evaporation rates under illuminated and dark conditions, net rates, and measured enthalpies. The water evaporation efficiencies for the AZC–CA/CA samples at 30, 40, and 50 wt% were determined to be 104.52%, 119%, and 104.64%, respectively. These values surpassed the efficiencies observed for pure water (19.63%) and the 30 wt% ZC–CA/CA evaporator (95.06%). As indicated by the surface roughness data (Figure S11, Supporting Information), the AZC–CA/CA exhibited a considerably greater average roughness (5.55 µm) than both CA (1.63 µm) and ZC–CA/CA (2.23 µm). This increased roughness is likely attributed to the dense and uniform decoration of AuNPs across the carbon framework. Such an increase in surface roughness promotes multiple light scattering and internal reflection, consequently increasing solar absorption and local heat generation. In contrast, the smoother surface of the ZC–CA/CA is more prone to reflection losses. In conclusion, the AZC–CA/CA hybrid evaporators outperformed the single‐component ZC–CA/CA evaporators owing to the synergistic effects stemming from broadband carbon absorption and plasmonic heating facilitated by the dense AuNPs.

Efficiencies for solar‐to‐vapor conversion that appear to exceed 100% do not violate the principle of energy conservation. Rather, these values arise from calculations that consider only the incident solar input. Such high efficiencies are a characteristic feature of photothermal evaporators possessing hydrophilic porous structures, particularly those based on cellulose. This enhanced efficiency is mainly attributed to 1) the reduced vaporization enthalpy of intermediate water found in confined capillaries and 2) minimized heat loss achieved through localized thermal confinement.^[^ ^48^, ^49^, ^50^, ^51^ ^]^ Subsequent investigations will involve further desalination and outdoor performance tests of the 40 wt% AZC–CA/CA evaporator.

**Figure 6:**
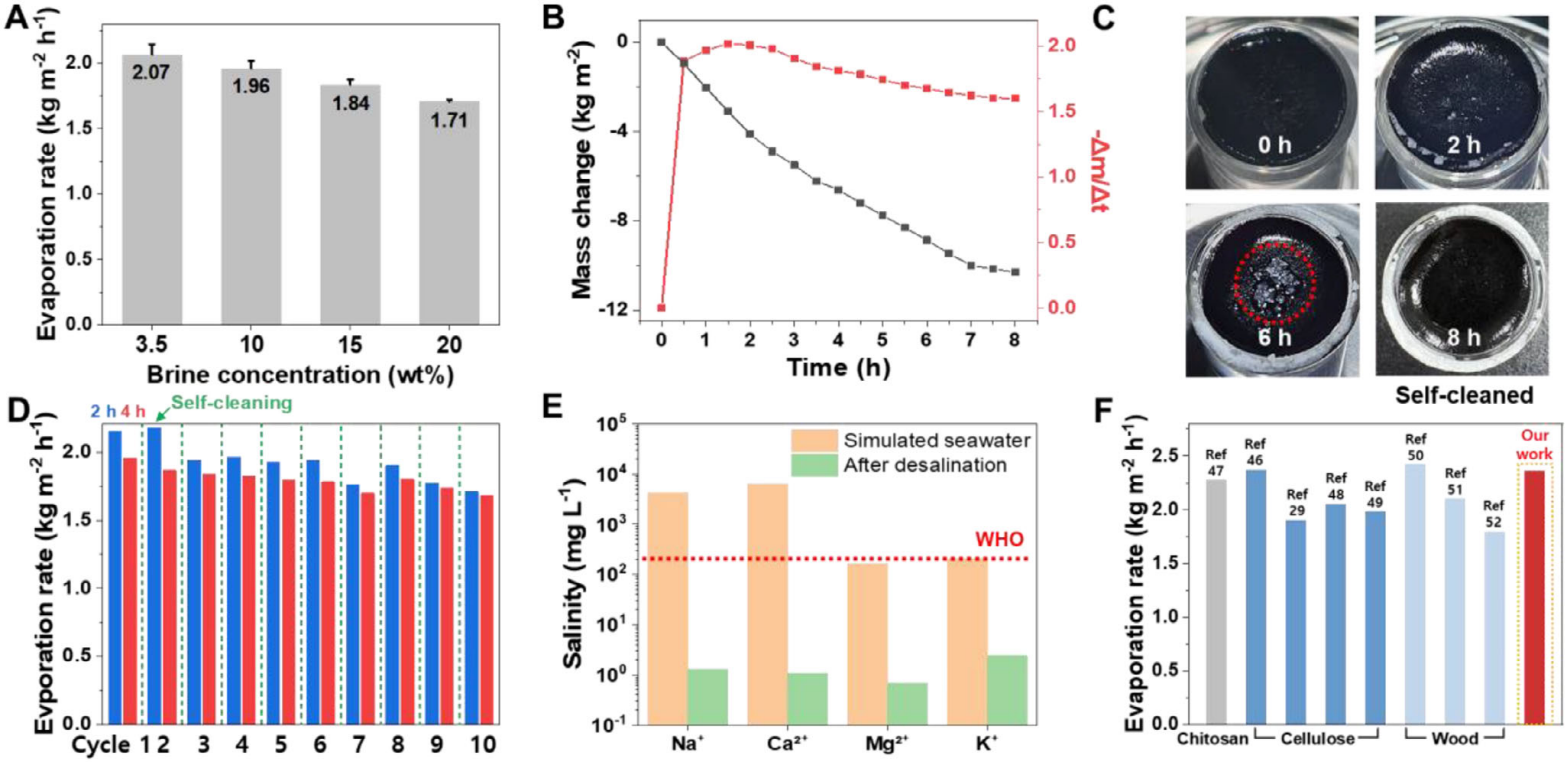
A) Effect of brine concentration on the evaporation rates (ν) using the AZC–CA/CA evaporator under 1 sun illumination for 1 h. B) Real‐time mass change (Δm) and corresponding mass loss rate (Δm/Δt) of the AZC–CA/CA evaporator during 8 h of operation at 3.5 wt% NaCl). C) Top‐down view of the AZC–CA/CA solar evaporator illustrating self‐cleaning performance after 6 h of illumination, followed by 8 h in a 20 wt% NaCl solution. D) Long‐term cycling stability was assessed over 10 desalination cycles (comprising 2 h and 4 h durations) at 3.5 wt% NaCl. E) Analysis of major ion concentrations (Na^+^, Ca^2+^, Mg^2+^, and K^+^) in simulated seawater before and after desalination. F) Performance comparison of evaporation rates under 1 sun illumination against other state‐of‐the‐art biomass‐based photothermal evaporators. The reference systems are grouped according to their primary photothermal material type, as indicated below the graph.

**Figure** **6**A shows that the AZC–CA/CA evaporator maintained high evaporation rates across a broad spectrum of brine concentrations, reaching 1.71 ± 0.02 kg m^−2^ h^−1^ even under saturated conditions (20 wt% sodium chloride (NaCl)). This exceptional stability can be attributed to its unique bilayer architecture: the vertically aligned CNF layer ensures a continuous water supply to the surface, while the photothermal AZC layer effectively confines heat locally. During an 8‐hour‐long‐term evaporation test in 3.5 wt% brine, the AZC–CA/CA evaporator showed a gradual decline in evaporation rate (−Δm/Δt) after ≈1.5 h, which indicates the beginning of salt accumulation on the evaporation surface (Figure 6B). Despite this, evaporation continued steadily throughout the full 8 h period, indicating that the evaporator maintained consistent desalination capabilities. Considerable salt deposition that accumulated after 6 h of operation under high salinity conditions (20 wt% NaCl) was effectively removed via salt redissolution, thereby demonstrating the evaporator's inherent self‐cleaning capability (Figure 6C). The evaporator's cyclability was subsequently assessed through ten repeated evaporation cycles that incorporated self‐cleaning steps at 3.5 wt% NaCl. In the initial cycle, the evaporation rates measured after 2 and 4 h were 2.15 and 1.96 kg m^−2^ h^−1^, respectively. Even after ten complete cycles, the rates remained at 1.71 and 1.68 kg m^−2^ h^−1^, corresponding to the retention of ≈86% of the initial evaporation rate after ten cycles. To assess internal morphology after long‐term cycling, the evaporator underwent a 30 min self‐cleaning process following the tenth cycle, then was frozen in liquid nitrogen and re‐freeze‐dried for SEM analysis. Cross‐sectional images revealed only slight salt accumulation compared with pristine microchannels, which was largely removed by self‐cleaning without evidence of channel blockage (Figure S12B, Supporting Information). Higher‐magnification images further showed minor salt deposits on some channel walls, whereas other regions appeared clean, and the AZC photothermal particles remained uniformly dispersed without aggregation (Figure S12C, Supporting Information). The consistent implementation of the self‐cleaning process after each cycle effectively restored the evaporation performance, confirming the system's long‐term durability and reusability under prolonged operating conditions (Figure 6D).

Using inductively coupled plasma optical emission spectroscopy (ICP‐OES) (Figure 6E), the ion content of the desalted water, obtained from simulated seawater evaporation, was analyzed. A substantial reduction was observed in the concentrations of major cations (Na^+^, K^+^, Mg^2+^, and Ca^2+^). For instance, the Na^+^ concentration decreased from 10 000 ppm to less than 30 ppm. Furthermore, the levels of all other analyzed ions also fell below the limits recommended by the World Health Organization for drinking water, confirming the evaporator's excellent desalination capability. A comparative analysis in Figure 6F reveals that our evaporator achieved exceptionally high evaporation rates, delivering superior or comparable performance relative to previously reported biomass‐based photothermal evaporator systems.^[^ ^32^, ^52^, ^53^, ^54^, ^55^, ^56^, ^57^, ^58^ ^]^ Previous cellulose‐based evaporators incorporating carbon photothermal materials have typically exhibited evaporation rates in the range of ≈1.82–1.95 kg m^−2^ h^−1^ under 1 sun.^[^ ^32^, ^52^ ^]^ In comparison, our AZC–CA/CA evaporator achieved 2.36 kg m^−2^ h^−1^, comparable to the best performing cellulose‐based systems. Notably, recent plasmonic hybrid carbon systems have demonstrated evaporation rates of ≈1.5–2.29 kg m^−2^ h^−1^ under 1 sun,^[^ ^59^ ^]^ positioning our system at the upper limit of this performance range. In summary, the evaporator exhibited outstanding evaporation performance, efficient salt rejection, and reliable self‐cleaning capability in both pure and saline water.

### 2.4 Real‐World Solar Evaporation, Large‐Scale Production, and Full Biodegradability

**Figure 7:**
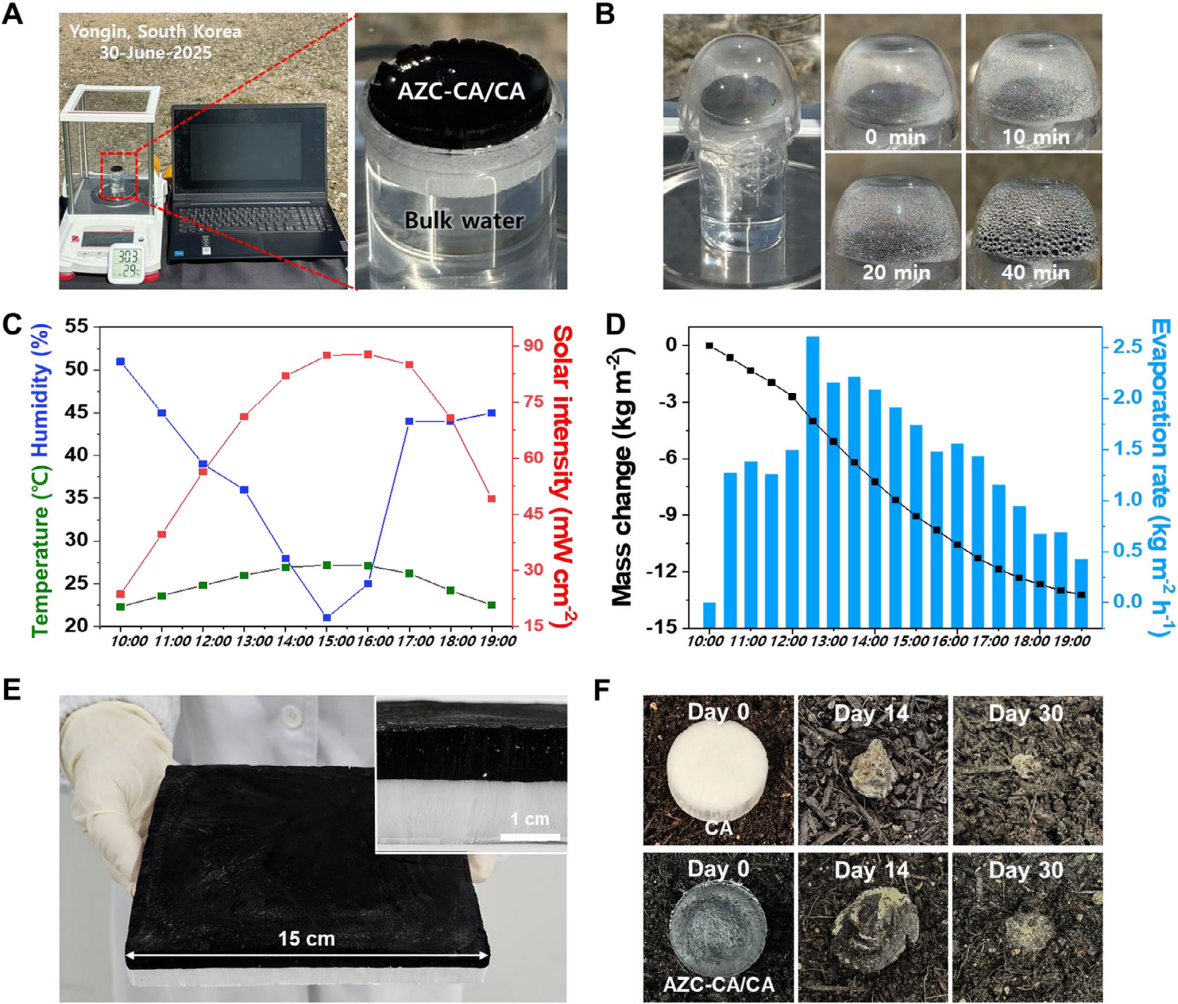
Outdoor solar evaporation performance and scalability of the AZC–CA/CA evaporator. A) Experimental setup for outdoor solar evaporation tests under natural sunlight conditions. B) Water condensation on the quartz dome at 0, 10, 20, and 40 min, indicating continuous evaporation. C) Real‐time measurements of ambient temperature, relative humidity, and solar intensity during the test. D) Diurnal evaporation rate profile from 10:00 to 19:00. E) Image of the scaled‐up (15 × 15 cm) AZC–CA/CA evaporator, demonstrating structural uniformity across the larger area (inset: side view highlighting the bilayer structure). F) Assessment of the biodegradability of CA and AZC–CA/CA samples after 30 days of outdoor exposure.

**Figure** **7**A shows the outdoor solar‐driven evaporation tests, which were performed under natural sunlight at Kyung Hee University on May 30, 2025, from 10:00 to 19:00. As shown in Figure 7B, the continuous generation of water droplets on the quartz dome visually confirmed continuous evaporation. The evaporation rate increased with rising solar intensity between 10:00 and 15:00, reaching its peak between 12:30 and 13:30. Thereafter, it gradually decreased from 14:00 to 19:00 (Figure 7C). Figure 7D shows that the evaporation rate closely followed the diurnal variations in solar intensity and temperature, while the cumulative water loss confirmed the evaporator's stable performance under real‐world conditions. As shown in Figure 7E, the fabrication process could be scaled up without defects, allowing for the production of large‐area evaporators (150 mm × 150 mm × 20 mm). This demonstrates their potential for practical freshwater production and large‐scale desalination applications. An assessment of biodegradability (Figure 7F) revealed that both CA and AZC–CA/CA samples exhibited clear biodegradation over 30 days under outdoor conditions, indicating that the incorporation of AZC did not impede this process. In contrast to synthetic polymers, these cellulose‐based materials exhibited complete decomposition.

## 3 Conclusion

This work presents the successful development of a fully bio‐based, water‐processable solar evaporator. Its design incorporates a cross‐linked CNF support integrated with a plasmonically enhanced, MOF‐derived carbon photothermal layer. Using sequential unidirectional ice templating, the evaporator exhibited vertically aligned porous channels, which ensure rapid water transport and efficient vapor release while exhibiting strong interfacial adhesion in the absence of binders. The in situ synthesis of AZC enabled both broadband light absorption and localized plasmonic heating, resulting in a high evaporation rate of 2.36 kg m^−2^ h^−1^ and an impressive efficiency of 119% under standard solar irradiation. These values surpass those of most cellulose‐based evaporators (typically 1.0–1.8 kg m^−2^ h^−1^ under 1 sun) and are comparable to, or even exceed, state‐of‐the‐art plasmonic hybrid carbon systems (typically 1.5–2.29 kg m^−2^ h^−1^ under 1 sun) recently reported. The developed system not only exhibited excellent performance in high‐salinity brines but also demonstrated robust cycling stability, a direct result of its self‐cleaning capability. Furthermore, the evaporator demonstrated complete biodegradability and was successfully fabricated on a large scale, confirming its environmental sustainability and practical applicability. Owing to these advantages, the developed solar evaporator shows strong potential for seawater desalination, emergency water supply in disaster‐stricken areas, and the provision of safe drinking water in remote or resource‐limited regions. These results underscore the potential of nanocellulose–plasmonic carbon hybrid materials for next‐generation solar desalination technologies.

## 4 Experimental Section

### Chemicals and Materials

Gold(III) chloride trihydrate (HAuCl_4_·3H_2_O), cellulose powder (C6288, cotton linters), TEMPO (98%), zinc acetate dihydrate (Zn(CH_3_CO_2_)_2_·2H_2_O), 2‐MeIm, and NaBH_4_ were obtained from Sigma–Aldrich (USA). Sodium bromide (NaBr), sodium hypochlorite solution (NaClO, 6%), hydrochloric acid (HCl), GA solution (C_5_H_8_O_2,_ 50%), sodium hydroxide (NaOH), NaCl, calcium chloride dihydrate (CaCl_2_⋅2H_2_O), magnesium chloride hexahydrate (MgCl_2_⋅6H_2_O), and potassium chloride (KCl) were purchased from Duksan Pure Chemicals Co. (South Korea). All chemicals were used as received without further purification.

### Preparation of ZC

Zn(CH_3_CO_2_)_2_·2H_2_O (30 g) was dissolved in 500 mL of distilled water, while 2‐MeIm (112.2 g) was dissolved separately in another 500 mL of distilled water, maintaining a molar ratio of 1:10. The two solutions were then combined under stirring and allowed to react for 5 min, during which the mixture gradually turned white. The resulting suspension was aged undisturbed at room temperature for 24 h. The precipitated ZIF‐8 particles were collected by centrifugation at 8000 rpm, thoroughly washed with distilled water and ethanol, and dried in an oven at 80 °C. The dried ZIF‐8 powder was carbonized under a nitrogen atmosphere (flow rate: 200 cc min^−1^) at 900 °C for 2 h, with a heating rate of 5 °C min^−1^, yielding ZC.

### Preparation of AZC

A total of 5 mg of ZC was dispersed in 5 mL of deionized water and sonicated for 30 min to obtain a uniform suspension. Then, varying volumes (0.5, 1.0, and 2.0 mL) of 0.01 m HAuCl_4_·3H_2_O solution were added under vigorous stirring, and the reaction was allowed to proceed for 2 h. Then, 400 µL of freshly prepared 0.35 m NaBH_4_ solution in deionized water was added dropwise to reduce the gold precursor. The mixture was stirred for an additional 2 h to ensure complete formation of AuNPs. The AZC particles were collected by centrifugation at 5000 rpm for 10 min and washed multiple times with deionized water to eliminate any remaining impurities. The final product was dried overnight in an oven at 60 °C for subsequent use. Samples prepared with 0.5, 1.0, and 2.0 mL of the gold precursor solution were designated as A_0.5_ZC, A_1_ZC, and A_2_ZC, respectively, where the number reflects the volume (mL) of HAuCl_4_·3H_2_O solution used during synthesis.

### Preparation of Cellulose Nanofiber Dispersion

CNF dispersions were prepared via a TEMPO‐mediated oxidation followed by high‐pressure homogenization, adapting previously reported methods with minor modifications. Briefly, 0.16 g of TEMPO and 1 g of NaBr were dissolved in 1 L of distilled water under stirring at 400 rpm. Cellulose powder (10 g) was then added. The oxidation was initiated by adding 62 g of NaClO solution, while the pH was maintained at 10 through continuous titration with 0.5 m NaOH. After the reaction was completed, the oxidized cellulose was washed repeatedly by centrifugation with distilled water until a neutral pH was reached. The TEMPO‐oxidized cellulose dispersion was then homogenized at 1000 bar for 10 cycles to produce individualized CNFs. Finally, the GA cross‐linked CNF suspension was adjusted to a concentration of 2.5 wt%.

### Preparation of Cross‐Linked Cellulose Nanofiber Aerogel

The pH of the as‐prepared CNF suspension was adjusted to 4.0 by adding 0.5 m HCl solution under continuous stirring. Then, a suitable amount of GA was introduced to the suspension while stirring at 450 rpm. The mixture was stirred for 1 h at room temperature, followed by heating at 80 °C for 3 h to facilitate cross‐linking. Finally, the resulting aqueous gel was degassed under vacuum to remove any trapped air bubbles. The GA‐cross‐linked CA was produced using a unidirectional ice‐templating method. In this process, 4 mL of the cross‐linked CNF dispersion was poured into a cylindrical plastic mold (25 mm diameter, 40 mm height) positioned on a copper plate cooled by direct contact with liquid nitrogen. The thermal gradient created by the liquid nitrogen induced unidirectional ice crystal growth throughout the dispersion. Once fully frozen, the samples were freeze‐dried overnight at −70 °C under 5 mTorr to yield the final CA. For comparison, a control cellulose aerogel without GA cross‐linking was also prepared, referred to as uncross‐linked CA.

### Fabrication of Bilayer Solar Evaporator

Bilayer‐structured solar evaporators were prepared by integrating the synthesized photothermal materials into the GA‐cross‐linked CNF dispersion. The fabrication of the ZC/cross‐linked CNF and AZC/cross‐linked CNF composite aerogels followed the same procedure as the pure cross‐linked CA, with the exception that photothermal materials (ZCs or AZCs) were added to the CNF matrix at loadings of 30, 40, or 50 wt% relative to the CNF weight. The designated amount of photothermal material was thoroughly mixed with the cross‐linked CNF dispersion and sonicated for 30 min to ensure a uniform dispersion. The water transport layer was prefrozen in the polystyrene (PS) template, after which 1 mL of photothermal material (ZC or AZC) integrated cross‐linked CNF gel was poured onto the frozen layer. Once the bilayer composite was fully frozen, the samples were dried at −70 °C under 50 mTorr pressure. Using the same method and an expanded PS template, a large‐scale solar evaporator measuring 150 mm × 150 mm × 20 mm was fabricated.

### Analysis of Solar Evaporation System

To evaluate the solar steam generation performance, the fabricated evaporator was placed on a microbalance while floating in a beaker filled with distilled water. The setup was then irradiated with a xenon‐lamp solar simulator (PEC‐L01, Peccell Technologies) at 1 sun intensity. The mass loss from water evaporation was continuously recorded.

Because evaporation can be affected by factors such as light intensity, ambient temperature, humidity, and atmospheric pressure, all experiments were performed under controlled conditions: a room temperature of 23.5 °C and relative humidity of 30%.

The evaporation rate (ν) was calculated using Equation (1):^[^ ^32^ ^]^

(1) $$v=\frac{\Delta m}{S\Delta t}$$

where Δm denotes the mass change over the evaporation duration (Δt), and S represents the surface area of the evaporator. The evaporation rate was determined as the average value obtained from at least three independent samples (n ≥ 3).

The solar‐to‐vapor conversion efficiency (η) was determined by calculating the ratio of the net energy used for water evaporation to the total incident solar energy under 1 sun irradiation. The net evaporation rate was derived by subtracting the mass change observed in dark conditions from that recorded under 1 sun illumination. The enthalpy of vaporization was experimentally measured using DSC. Solar energy input was calculated based on the optical concentration factor and solar irradiance. Intermediate water content was determined from DSC thermograms (−40 to 20 °C) by integrating the endothermic melting peak and calculated by utilizing Equations ((2), (3), (4)):^[^ ^60^ ^]^

(2) $$W_{\mathrm{total}}=\frac{m_{\mathrm{wet}}-m_{\mathrm{dry}}}{m_{\mathrm{wet}}}$$

(3) $$W_{\mathrm{free}}=\frac{\Delta H_{\mathrm{sample}}}{\Delta H_{\mathrm{ice}}}\;$$

(4) $$W_{\mathrm{int}+\mathrm{nf}}=W_{\mathrm{total}}\;-W_{\mathrm{free}}$$

where *m*_wet_ and *m*_dry_ represent the masses of the wet and dry samples, respectively. *W*_total_ denotes the total water fraction (g g^−1^). Δ*H*_sample_ is the melting enthalpy obtained by integrating the DSC endothermic peak (J g^−1^) and Δ*H*_ice_ is the enthalpy of fusion of pure ice (334 J g^−1^). Thus, *W*_free_ represents the free water fraction. *W*_int + nf_ represents the combined fraction of intermediate and non‐freezable water (g g^−1^).

The efficiency was then calculated according to Equation (5):^[^ ^61^ ^]^

(5) $$\eta =\frac{v_{net}\times h_{vap}}{C_{opt}\times P}\times 100\%$$

where *v_net_* represents the net evaporation rate (kg m^−2^ h^−1^), *h_vap_* is the measured enthalpy of vaporization (J g^−1^), *C_opt_* is the optical concentration (set to 1 under 1 sun), and *P* denotes the solar irradiance (1000 W m^−2^).

Desalination experiments were performed using brine solutions of varying concentrations (3.5, 10, 15, and 20 wt%). For long‐term performance assessment, a continuous desalination test was performed with 3.5 wt% brine (simulated seawater) over 8 h. During each cycle, the evaporation rate in simulated seawater was recorded at 2 and 4 h intervals. A self‐cleaning process with a 1‐h rest period was integrated between cycles to ensure system stability. To assess practical application, outdoor evaporation tests were performed under natural sunlight on May 30, 2025, at Kyung Hee University. The solar evaporator was exposed to sunlight from 10:00 AM to 7:00 PM local time to evaluate its performance under real outdoor conditions.

### Characterizations

The morphologies of AZC and AZC–CA/CA were analyzed using FE‐SEM (Merlin, Carl Zeiss). The uniform growth of AuNPs on ZC was further confirmed by FE‐TEM (JEM‐2100F, JEOL) along with corresponding EDS mapping images. Particle size distribution was evaluated by measuring the hydrodynamic diameters of ZIF‐8, ZC, and AZC particles using a DLS analyzer (Nano‐ZS90, Malvern Panalytical). Specific surface areas for these particles were determined via BET analysis (ASAP 2020, Micromeritics). Chemical compositions were examined using FT–IR spectroscopy (Nicolet iN10 MX, Thermo Fisher). The crystalline phases of the samples were examined using XRD analysis (SmartLab, Rigaku). Thermal conductivity was measured with a hot disk thermal constants analyzer (Hot Disk TPS‐3500). Raman spectra of ZIF‐8, ZC, and AZC were obtained using a QE‐PRO spectrometer (Ocean Optics Inc.). Surface wettability was assessed by static contact angle measurements using a contact angle meter (Attension Theta Optical Tensiometers, Biolin). Optical absorption properties were recorded using an UV–vis–NIR spectrometer (Cary 5000, Agilent Technologies), and the absorbance spectrum A(𝜆) was calculated using Equation (6):^[^ ^32^ ^]^

(6) $$\alpha =\frac{\int_{300nm}^{2500nm}I_{s}\left(\lambda \right)A\left(\lambda \right)d\lambda }{\int_{300nm}^{2500nm}I_{s}\left(\lambda \right)d\lambda }$$

Incorporating the AM 1.5 solar spectral irradiance profile as defined in ISO 9845‐1 (1992), surface roughness profiles were acquired using a surface profiler (Dektak 150, Bruker). The enthalpy of water evaporation was determined with a DSC 250 (TA Instruments) at a heating rate of 10 °C min^−1^ across a temperature range of 5–160 °C. Concentrations of residual ions in condensed water, collected after evaporating simulated seawater via the photothermal evaporator, were analyzed using ICP‐OES (iCAP PRO, Thermo Fisher). Water uptake was evaluated by immersing samples in water for 3 h, followed by weighing, and calculating the water uptake percentage according to Equation (7):^[^ ^32^ ^]^

(7) $$Wateruptake\;\;\left(\;\%\;\right)=\frac{\left(m_{\mathrm{wet}}-m_{\mathrm{dry}}\right)}{m_{\mathrm{dry}}}\;\;\times 100$$

where *m*_wet_ and *m*_dry_ are as previously defined.

The biodegradability of the evaporator was evaluated by burying samples in soil‐filled pots with drainage, which were then placed outdoors at Kyung Hee University starting June 3, 2024.

## Conflict of Interest

The authors declare no conflict of interest.

## Supporting information



Supporting Information

## Data Availability

The data that support the findings of this study are available from the corresponding author upon reasonable request.
