# Supplementary material for: Nanocellulose–MOF‐Derived Carbon Hybrid Aerogels with Hierarchical Micro/Nanostructures for Solar‐Driven Water Evaporation
Source: Adv Sci (Weinh). 2025 Oct 24;13(1):e16158. doi: 10.1002/advs.202516158 (PMC12767125; doi:10.1002/advs.202516158)
Supplement: Supplementary file 1 — Supporting Information [file ADVS-13-e16158-s001.docx]

Supporting Information

**Nanocellulose–MOF-Derived Carbon Hybrid Aerogels with Hierarchical Micro/Nanostructures for Solar-Driven Water Evaporation**

Suji Lee^1^, Kangyun Lee^1^, Youngho Jeon^1^, Yuri Seo^1^, Seohyun Park^1^, Youngsang Ko^1,2,^*, and Jungmok You^1,^*

^1^Department of Convergent Biotechnology & Advanced Materials Science, BK21 Interdisciplinary Program in IT-Bio Convergence System and Graduate School of Green-Bio Science, Kyung Hee University, 1732 Deogyeong-daero, Giheung-gu, Yongin-si, Gyeonggi-do 17104, South Korea

^2^NanoScience Technology Center, University of Central Florida, Orlando, FL 32826, USA

*Corresponding author: Youngsang Ko and Jungmok You

E-mail: Youngsang.Ko@ucf.edu (Y. Ko); jmyou@khu.ac.kr (J. You)


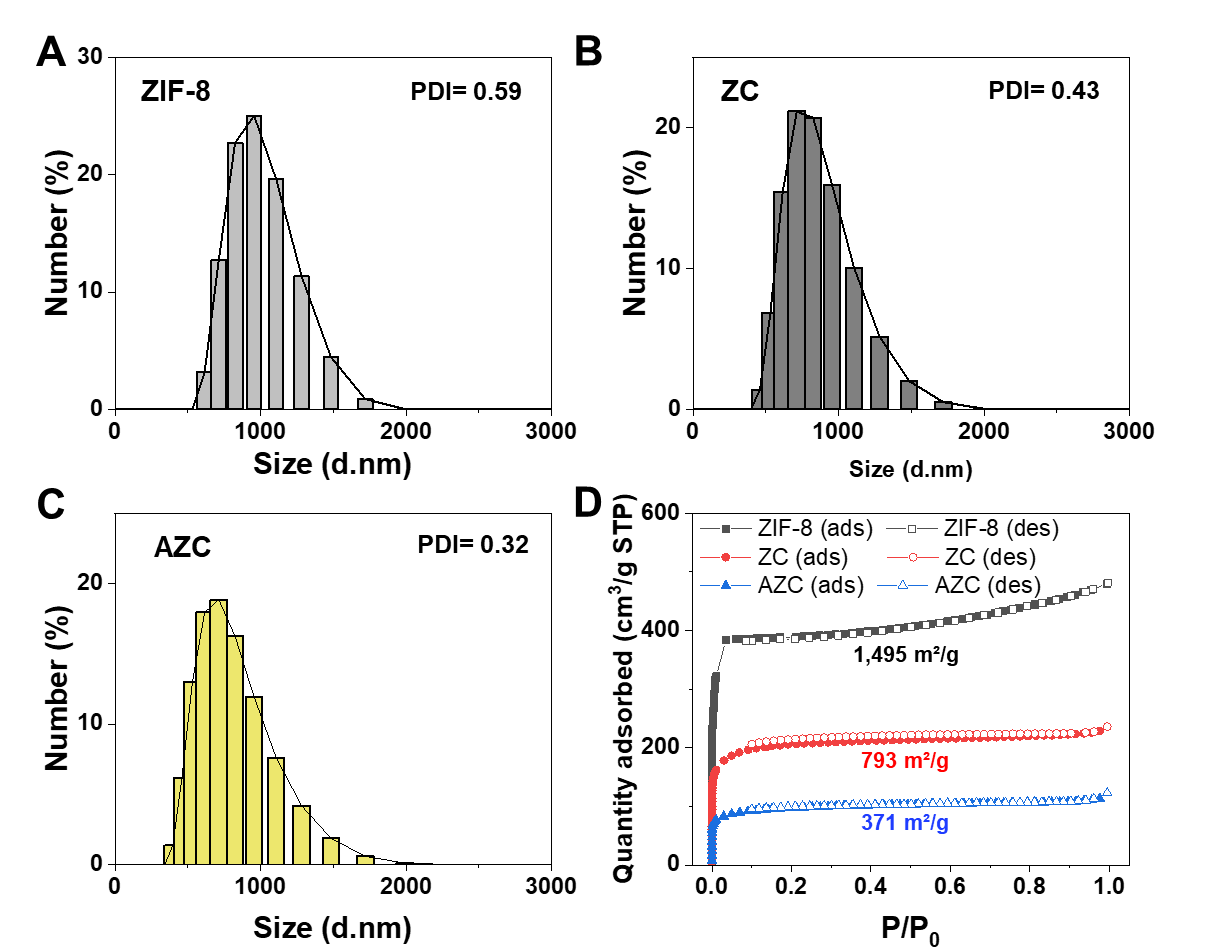


**Figure S1**. DLS analysis of (a) ZIF-8, (b) ZC, and (c) AZC nanoparticles, indicating average hydrodynamic diameters and particle distribution profiles. (d) N_2_ adsorption/desorption isotherms at of ZIF-8, ZC, and AZC.


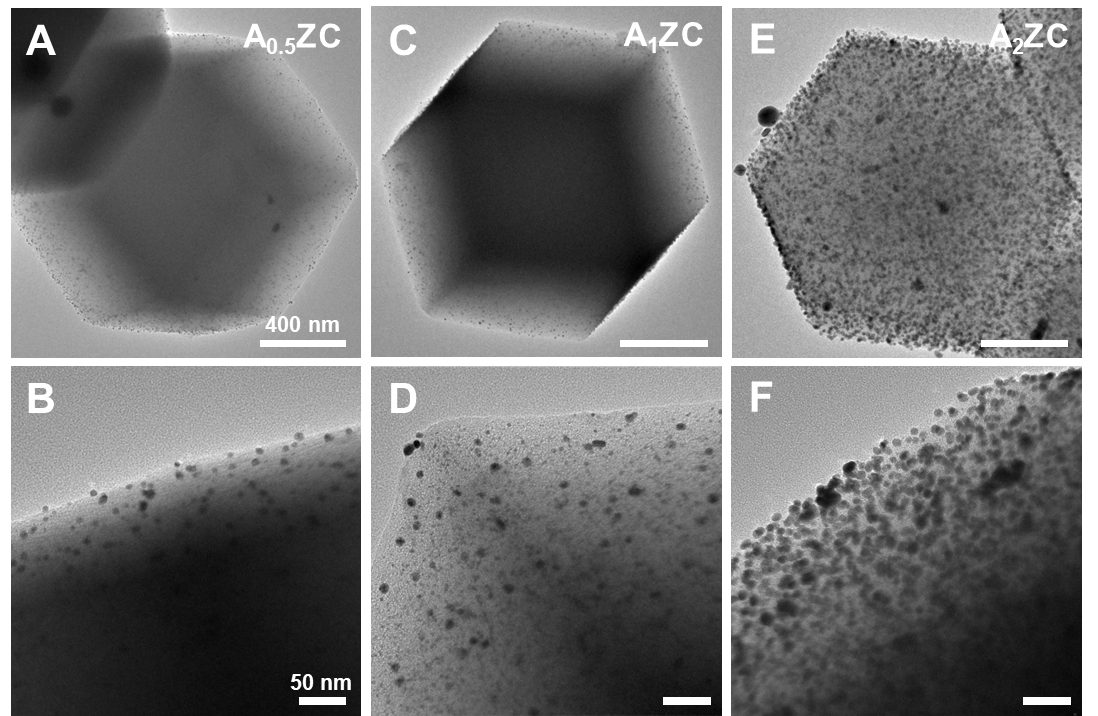


**Figure S2**. FE-TEM images of A_x_ZC powders (x = the amount of Au precursor solution used during the AuNPs loading). (A, B) A_0.5_ZC, (C, D) A_1_ZC, and (E, F) A_2_ZC.
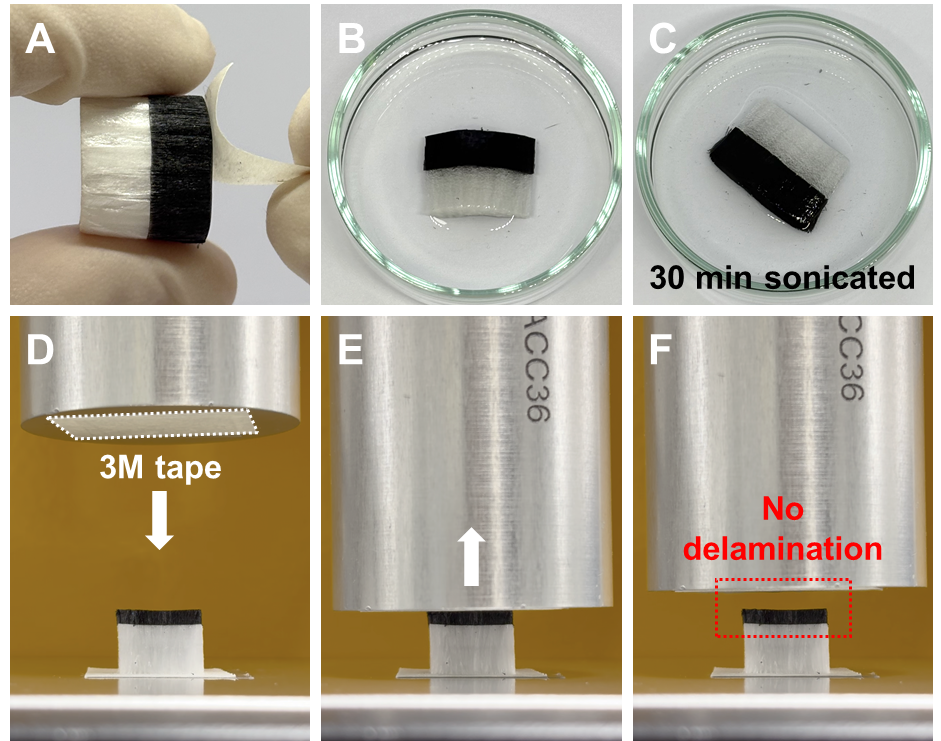


**Figure S3**. Adhesion and structural stability of the AZC–CA/CA evaporator. (A) Taping test in which 3M tape was applied to the photothermal layer and manually peeled off. (B) Evaporator immersed in water and (C) after 30 min of sonication. (D–F) Compression test using auxiliary tape to verify the adhesion stability between the photothermal layer and the water transport layer.


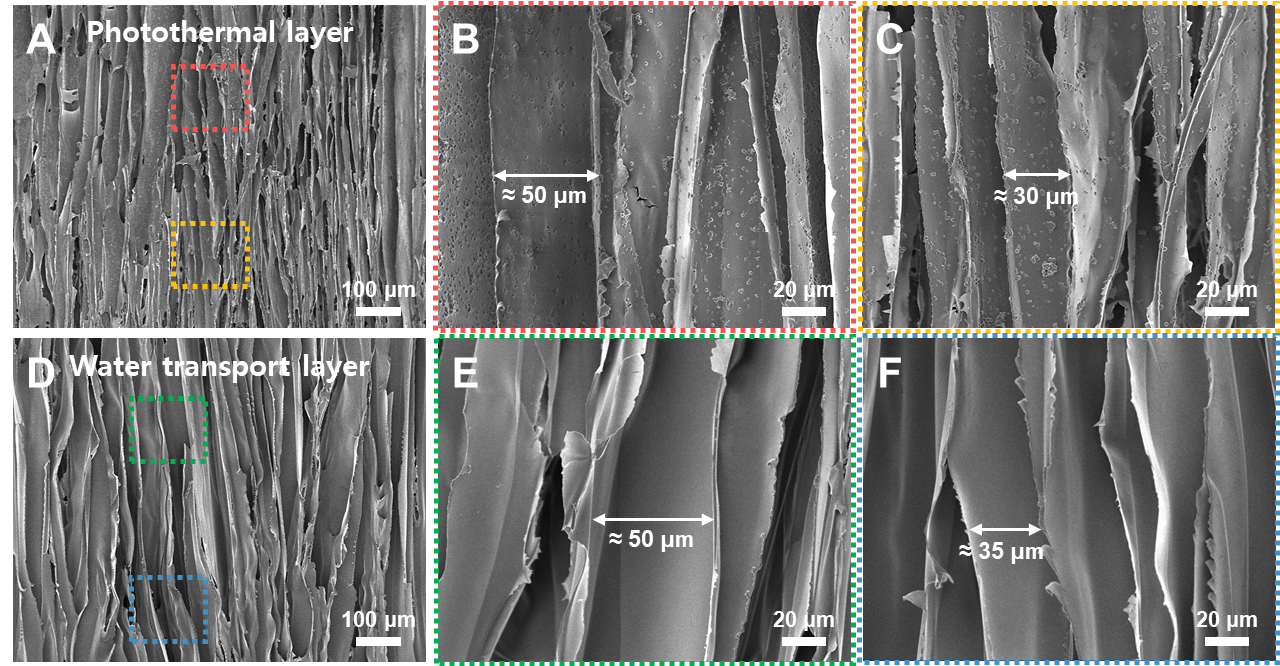


**Figure S4**. Cross-sectional SEM images of (A) AZC–CA photothermal layer and (D) CA water-transport layer, where (B) the top region of the photothermal layer, (C, E) the interfacial region between the two layers, and (F) the bottom region of the CA layer.


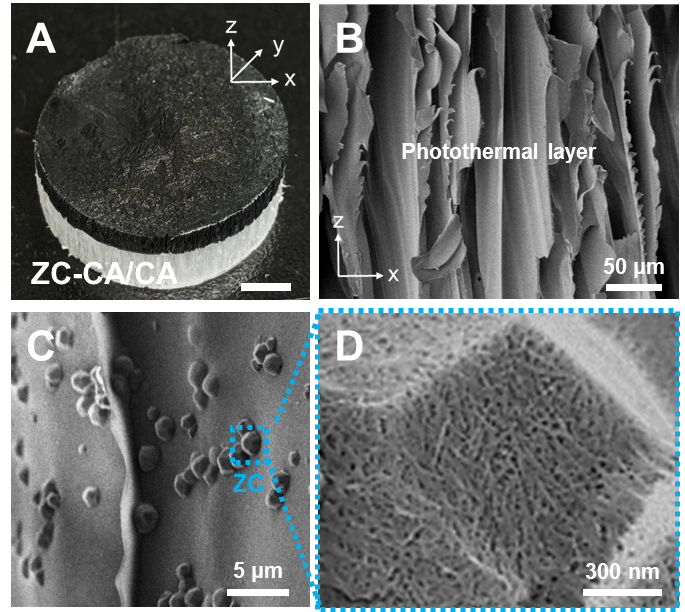


**Figure S5**. Photograph of (A) ZC-CA/CA evaporator and (B-D) cross-sectional SEM images of photothermal layer in corresponding sample.


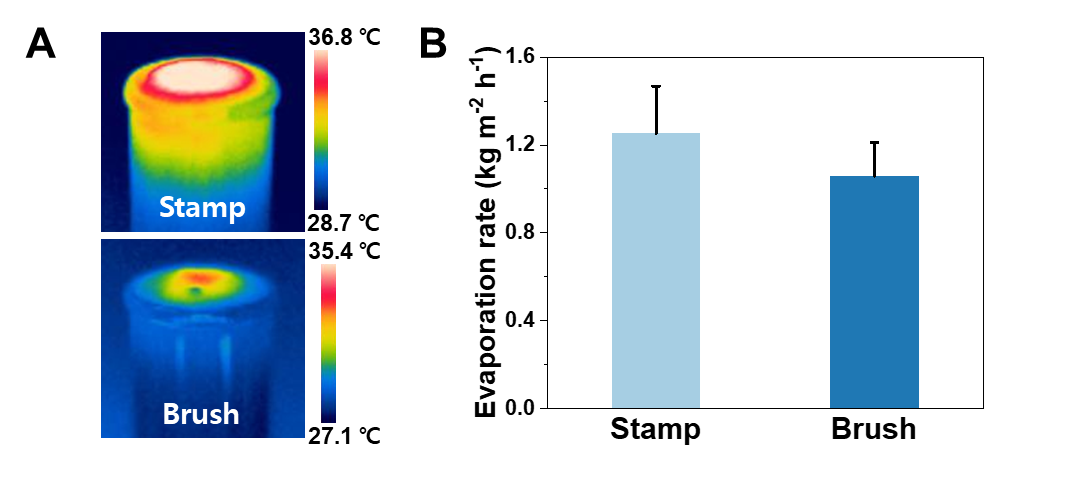


**Figure S6**. (A) Infrared (IR) thermal images and (B) evaporation rates of AZC-CA/CA evaporators fabricated by conventional stamping and brush coating. All solar vapor generation measurements were performed under 1 sun illumination.


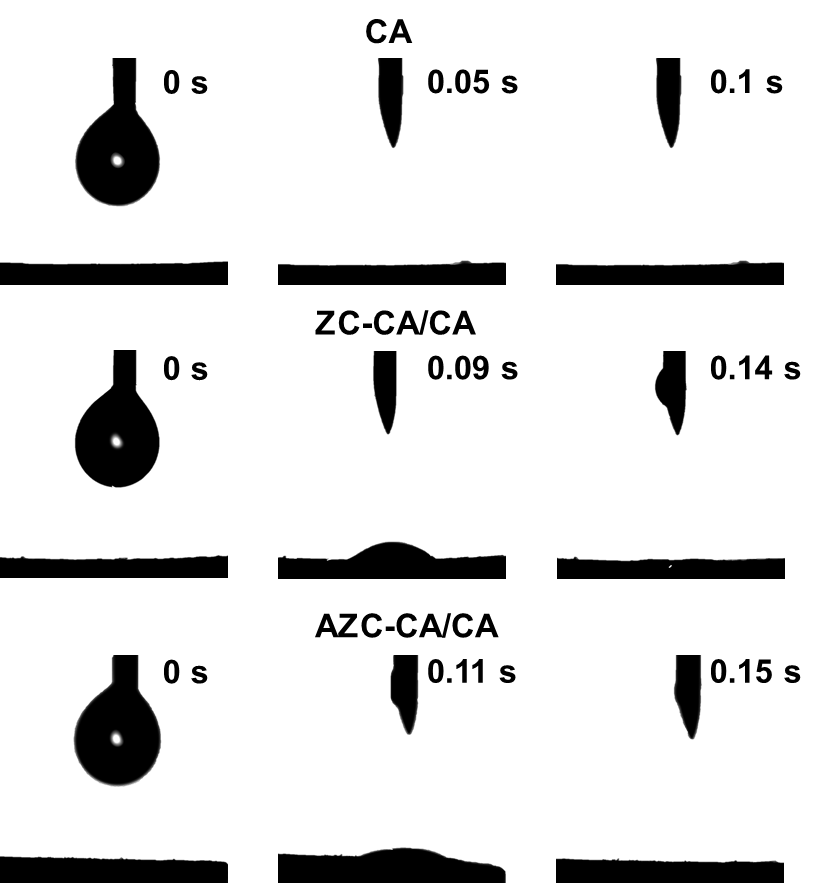


**Figure S7**. Water wettability behavior of CA, ZC-CA/CA, and AZC-CA/CA.


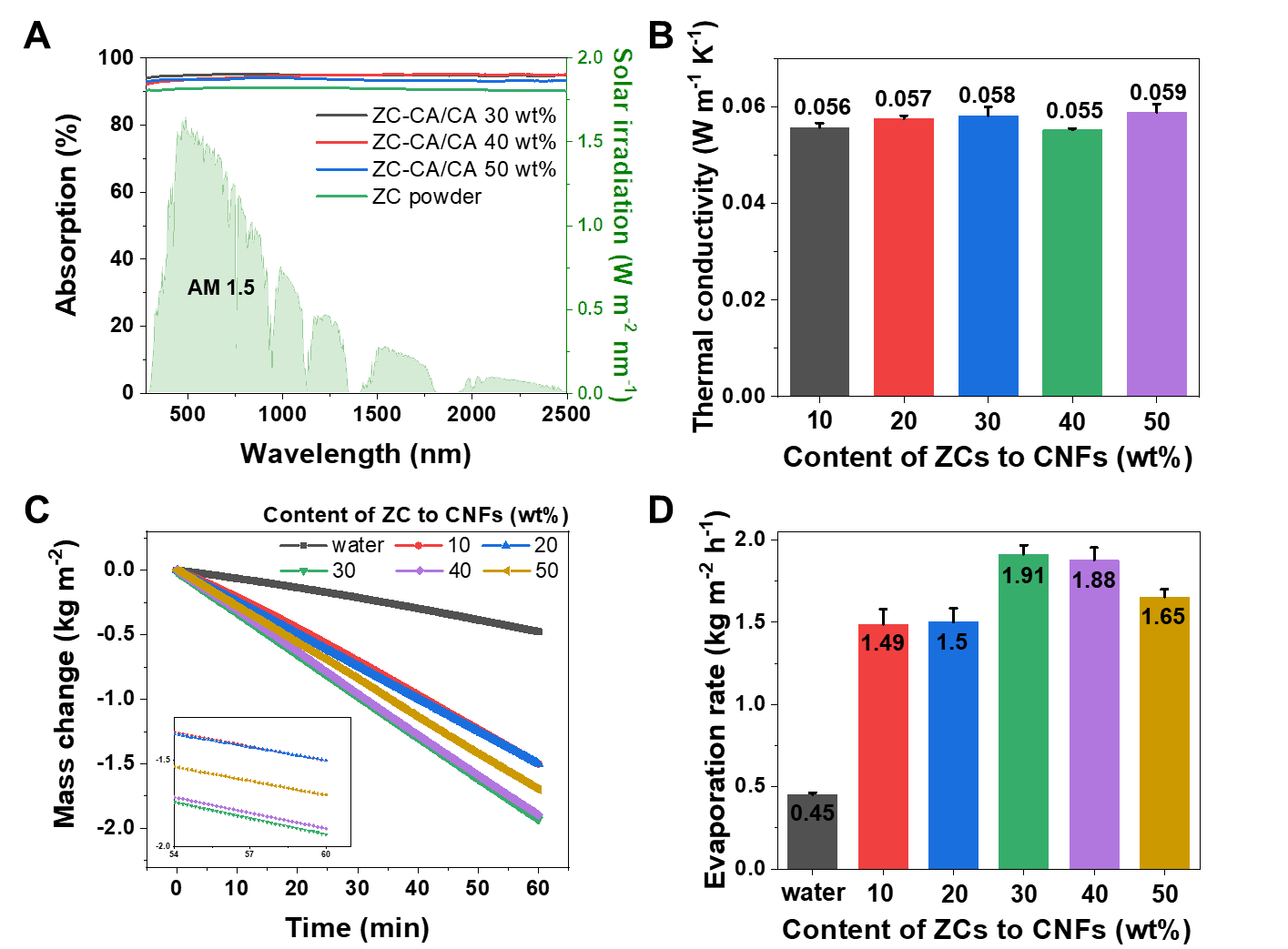


**Figure S8**. (A) UV-vis-NIR absorption spectra of pure ZC powder and ZC–CA/CA evaporators containing 30-50 wt% of ZC. (B) Thermal conductivities, (C) mass change, and (D) evaporation rates of ZC–CA/CA evaporators containing 10-50 wt% of ZC under 1 sun illumination.

**Figure S9**. Intermediate water analysis of AZC-CA/CA and ZC-CA/CA through DSC analysis.


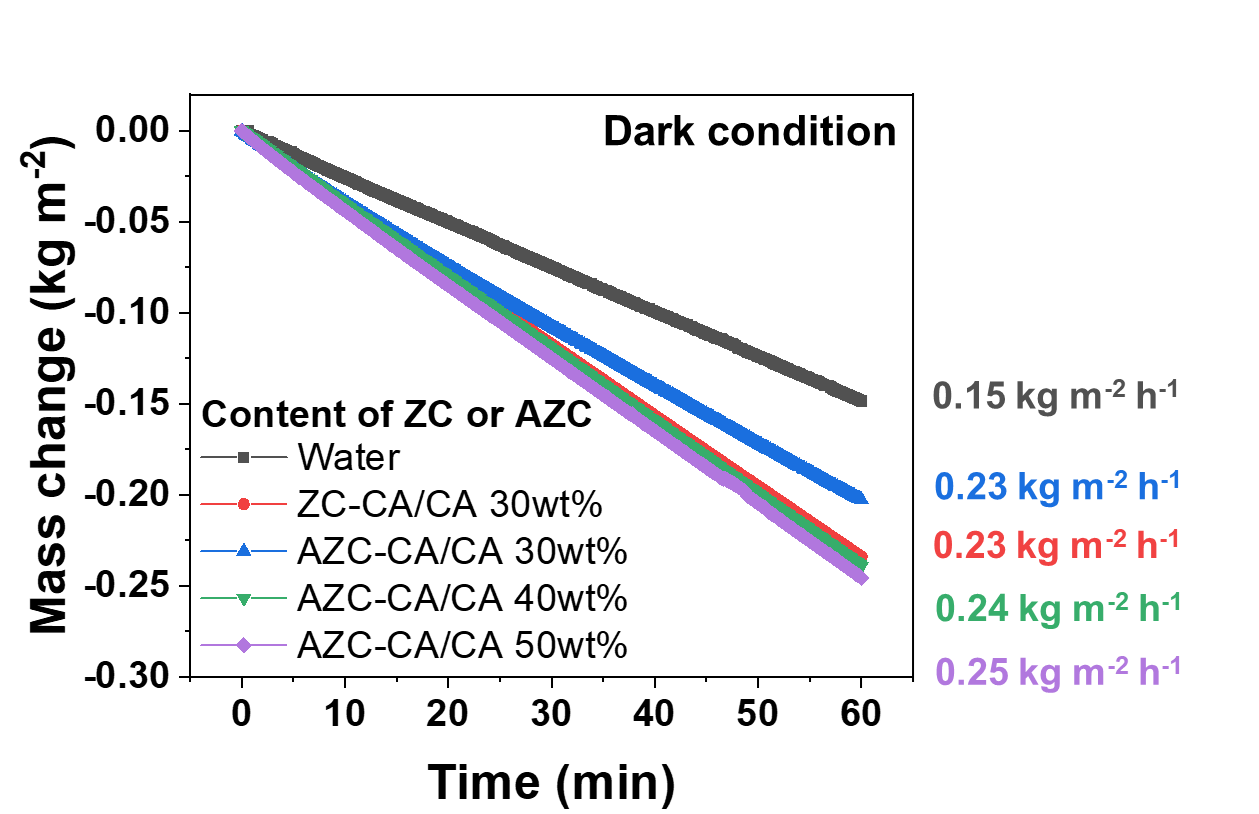


**Figure S10**. The mass change of water from the ZC-CA/CA and AZC-CA/CA evaporator with 30, 40, 50 wt% of ZC or AZC under dark condition.


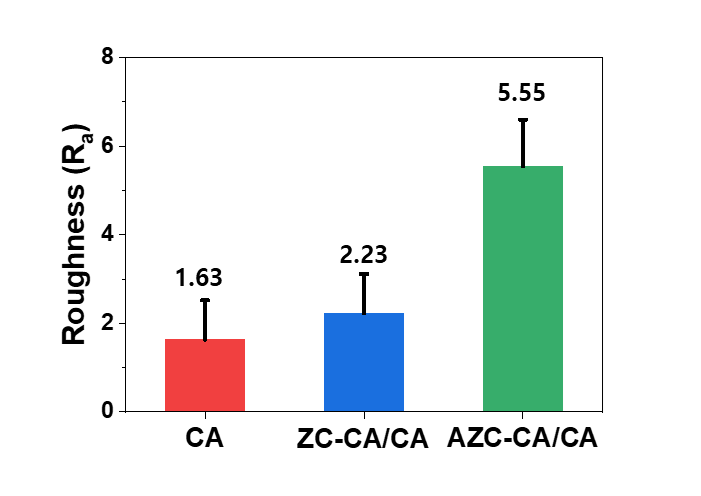


**Figure S11**. The surface roughness of CA, ZC-CA/CA and AZC-CA/CA.


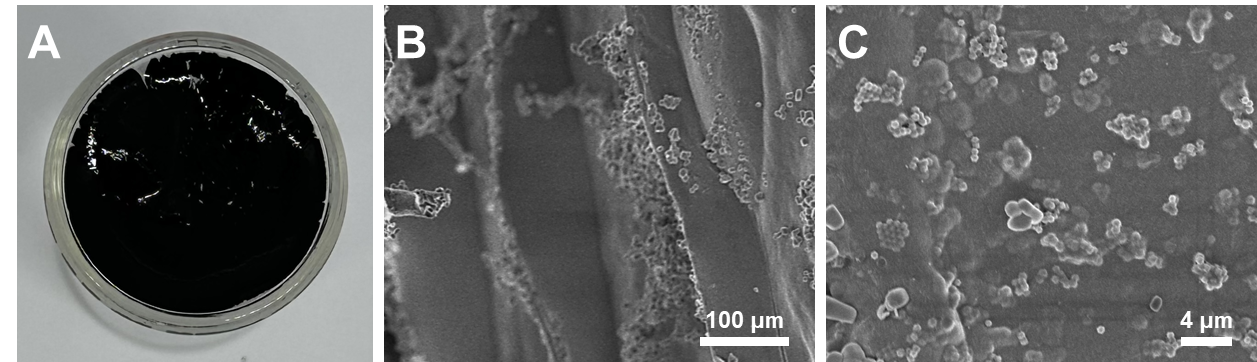


**Figure S12**. (A) Top-view photograph of the AZC–CA/CA evaporator after 10 desalination cycles in 3.5 wt% brine under 1 sun illumination, followed by 4 h of evaporation and then 30 min of self-cleaning. (B) Cross-sectional SEM image of the same sample, and (C) magnified image highlighting the microchannel surface morphology after long-term cycling.

**Table S1.** List of numerical values used to calculate evaporation efficiency.

| **Sample** | ***v*_light_**  **(kg m⁻² h⁻¹)** | ***v*_dark_**  **(kg m⁻² h⁻¹)** | ***v*_net_**  **(kg m⁻² h⁻¹)** | ***h*_vap_**  **(J g⁻¹)** | **Efficiency**  **(%)** |
| --- | --- | --- | --- | --- | --- |
| Water | 0.47 | 0.15 | 0.32 | 2203.5 | 19.63 |
| ZC-CA/CA 30 wt% | 1.91 | 0.23 | 1.68 | 2037 | 95.06 |
| AZC-CA/CA 30 wt% | 2.01 | 0.23 | 1.78 | 2124.6 | 104.52 |
| AZC-CA/CA 40 wt% | 2.36 | 0.24 | 2.12 | 2020.6 | 119 |
| AZC-CA/CA 50 wt% | 2.05 | 0.25 | 1.8 | 2091.5 | 104.64 |
